# Re-Evaluating the Stability of Al_2_O_3_ Barriers Prepared by Atomic Layer Deposition under Electrochemical Conditions

**DOI:** 10.1021/acsami.5c11388

**Published:** 2025-08-19

**Authors:** Andrew J. Bagnall, Ziwen Zhao, Mun Hon Cheah, Alina Sekretareva

**Affiliations:** Department of Chemistry—Ångström Laboratory, 8097 Uppsala University, Uppsala 75120, Sweden

**Keywords:** atomic layer deposition, electrochemical impedance spectroscopy, cyclic voltammetry, aluminum oxide, electronic tunnelling, insulation barrier

## Abstract

Atomic layer-deposited (ALD) films are widely used as insulating barriers in (photo)­electrochemical systems, yet their stability and charge-transfer behavior under operational conditions remain poorly characterized. Here, we systematically investigate how film thickness and electrolyte composition influence the performance of ALD-grown amorphous Al_2_O_3_ films on indium tin oxide. Using cyclic voltammetry and electrochemical impedance spectroscopy, we find that a thickness of ∼4–5 nm is required to achieve stable insulation and tunneling-limited electron transfer, which is significantly more than the minimum needed to form a continuous film. Moreover, the extracted tunneling decay constant, 0.30 Å^–1^, is lower than values reported for crystalline Al_2_O_3_, indicating noticeable charge transport through amorphous thin films. On the other hand, a reduction in the effective diffusion of redox active molecules at the electrode surface is suggested for films thicker than 3 nm. We further demonstrate that specific ions strongly influence film lifetime. Unexpectedly, we found that acetate buffers are significantly less detrimental to film stability compared to commonly used phosphate buffers. Moreover, the addition of low concentrations of Al^3+^ ions dramatically delays film degradation. In contrast, pH effects between 4 and 8 are minimal. Notably, film failure shows stochastic behavior while also being broadly consistent with gradual homogeneous dissolution rather than discrete pinhole formation previously reported for TiO_2_ and Al_2_O_3_ insulating films. These results reveal the critical and previously underappreciated role of electrolyte composition in determining the lifetime of insulating oxide films. Our findings offer practical design guidelines and highlight the need for controlled conditions when implementing ALD barriers in electrochemical devices.

## Graphical abstract

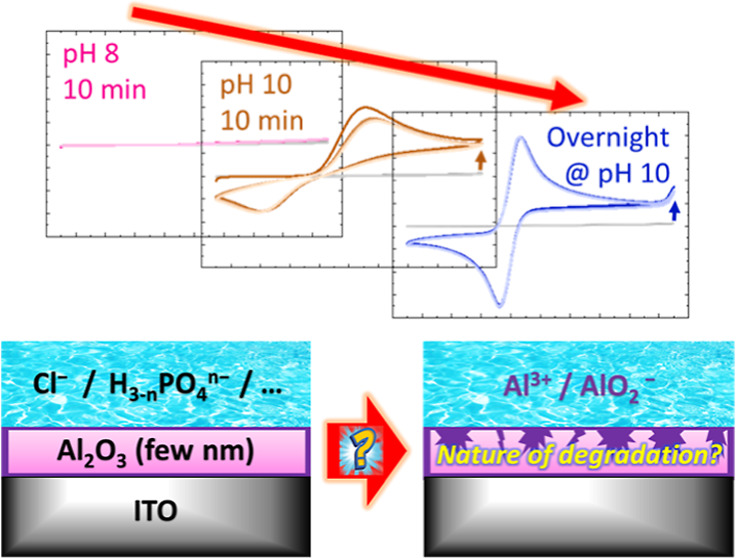

## 1 Introduction

Across a broad and diverse array of applications, there is a common demand for effective and stable nanoscale insulating barriers. In recent times, thin films of insulating metal oxides have been widely employed, most famously in microelectronics, including in the metal-oxide-semiconductor field-effect transistors,^1^ in anticorrosion coatings,^2,3^ photovoltaics,^4–6^ analytical electrochemistry,^7,8^ electrocatalysis,^9^ photocatalysis,^10^ and photoelectrocatalysis.^11–18^ Moderately thick films of a few to several nanometers may enable mediation of the current flow by sufficiently, but not completely, reducing the tunnelling probability with the effect of delaying the equilibration of electron populations. On the other hand, depending on its bandstructure, a semiconductive film may selectively facilitate the passage of one type of carrier for which the energetic barrier is lower, selectively allowing charge hopping through accessible states or easier direct tunnelling. Harnessing such long-range electron transfer has long been a major goal in chemistry;^19–21^ in photoelectrocatalysis, this naturally presents many potential opportunities for selectively manipulating the rates of charge transfer processes to encourage charge separation and disfavor recombination.^11^ Furthermore, the formation of inversion layers at the interface of semiconductor substrates with metal oxides can facilitate the transfer of photoexcited carriers by increasing their concentration near the surface and modulating their potentials to match the bandstructure or orbitals of a catalyst.^22^

Within this scope, a number of materials have been the subject of great scientific interest in the past decades, the most prominent examples being self-assembled monolayers (SAMs) and metal oxides. Although SAMs are widely studied, they are prone to pinhole formation, defects and instability.^8,23–25^ Hence, metal oxides have seen wider use in practical applications, especially due to their own versatility and wide range of deposition methods. This includes sol–gel processes, electrodeposition, various physical vapor deposition methods, such as sputtering, and chemical vapor deposition.^16^ However, control over film thickness, quality and homogeneity through these classical methods can be insufficient on the nanoscale for highly sensitive applications. Within this context, atomic layer deposition (ALD) has come to be seen as a particularly promising strategy to overcome these limitations.

Al_2_O_3_ grown from trimethylaluminum (TMA) and water has long been considered a model ALD process for its reliability under relatively mild conditions, producing highly insulating layers which are thermodynamically stable across a wide range of potentials at neutral pH values.^26,27^ In particular, its suitability for low-temperature ALD, compatible with temperature-sensitive substrates, should broaden its scope of application,^4,28^ making it a key potential candidate for tunnelling barriers in photoelectrodes, but this has thus far been hindered by underexplored stability issues, attributed to surface restructuring, with specific ionic species suspected to play a role.^29,30^ Al_2_O_3_ deposited by standard ALD procedures is generally amorphous, but can be converted, in part or in whole, to crystalline phases by annealing at temperatures above 800 °C or depositing under more aggressive conditions.^3,31–33^

The Pourbaix diagram for bulk aluminum gives a rough first estimation of the pH and potential range over which ALD Al_2_O_3_ layers can reasonably be employed, before considering the effects of other species in solution. However, the ability of other ions to bind and complexate aluminum or otherwise catalyze breakdown and heterogenisation of thin films is a crucial concern, as supporting electrolytes and buffers are essential for electrochemical studies.^34^ Chloride is known to be corrosive to metallic aluminum, causing pitting by penetrating the protective Al_2_O_3_ layer at neutral conditions,^27,35^ but its effects on Al_2_O_3_ thin films themselves remain debated in the literature. Many articles have implicated chloride as playing a role in accelerating film degradation,^2,30,36–39^ and consequently, most of the blame for the unexpectedly poor stability of Al_2_O_3_ films in neutral phosphate-buffered saline (PBS) has been attributed to its presence. A study by Kim et al. in fact observed that phosphate effectively counteracted the effect of chloride in their system, attributed to the formation of insoluble aluminum phosphates.^30,40^ On the contrary, Willis et al. implicated phosphate as the primary culprit in their system, and noted that stability over the period of several days was actually enhanced by the presence of chloride compared to deionized water, as deionized water alone induced hydration of the film, causing thickening and roughening, an observation shared by Correa et al.^3,29^

Kim et al.^30^ proposed two fundamental mechanisms for ALD film dissolution: Either the film dissolves evenly across its whole area, without defects and pinholes forming, or the dissolution occurs unevenly, generating defects and pinholes around which further dissolution is accelerated, perhaps in some ways comparable to chemically or electrically induced breakdown of Al_2_O_3_ layers on aluminum metal.^35,36^ To investigate this, their group developed an electrochemical methodology for studying the effect of aging in solution on ALD layers deposited on conductive indium tin oxide (ITO) glass over periods of up to a few days. By comparative analysis of capacitance^41^ and TEM data, they concluded that the dissolution at neutral pH was uneven and did generate pinholes.

Altogether, these studies broadly observed the expected rapid dissolution of unannealed Al_2_O_3_ in strong base and strong acid, in some cases with notably slower dissolution in acid than in base.^3,29,42^ However, at near-neutral pH, reported film behaviors vary significantly across studies, suggesting the presence of previously unrecognized factors that influence film stability under these conditions. These discrepancies likely stem from differences in experimental design and evaluation methods, making it difficult to draw general conclusions. Notably, while ALD-grown amorphous oxides are increasingly used as tunneling barriers and protective coatings, there is limited quantitative data on their charge-transfer characteristics and degradation across relevant electrochemical environments. Furthermore, no systematic comparisons have been made regarding the role of buffer composition in film stability.

In this study, we address these gaps by systematically evaluating both the charge-transfer properties and chemical stability of Al_2_O_3_ ALD films of varying thicknesses, focusing in particular on 5.0 nm films in a range of buffered solutions across the central pH scale. This allows us to assess their applicability in short-term (photo)­electrocatalytic studies where high tunneling barriers can be used to control carrier transport while protecting the electrode surface.^11^

## 2 Experimental Section

### 2.1 Chemicals and Materials

Ferrocenemethanol, buffer salts, acid solutions and potassium chloride were all acquired from commercial sources (Merck, FisherSci, VWR) in high purity. Deionized water was used in the preparation of all solutions. Vapor reagents in ALD were provided by MyFab: Deionized water and TMA (EpiValence, electronic grade). Reference electrodes were commercial (CH Instruments, Inc.). Graphite rod counter electrodes were sourced from ProGraphite GmbH and polished. ITO glass was sourced from Zhuhai Kaivo Optoelectronic Technology Co., Ltd. (001 Series), with the following specifications: Average flatness ≤0.38 μm/20 mm; sheet resistance of 7.5–8.3 Ω/sq; ITO thickness of 1780–1860 Å.

The main electrochemical stability measurements were taken using a Gamry 620 potentiostat. Measurements carried out as part of the EIS study were taken using an Ivium CompactStat. EIS experiments were carried out inside a faraday cage. In all cases, magnetically insulated wires were used for the connections from the potentiostat to the electrodes.

### 2.2 Deposition of Al_2_O_3_ Layers

Electrode samples were prepared with conductive ITO glass as the substrate. Glass slides were cut to 1.1 cm × 1.4 cm rectangles. Glass slides were cleaned by sonication in soapy water, deionized water and ethanol for 5 min each before drying with clean air immediately before atomic layer deposition, to minimize the probability of dust or contaminants on the surface.

Atomic layer deposition to deposit Al_2_O_3_ was carried out using a Picosun R-200 with TMA and H_2_O as precursors at 200 °C, with a 1 h temperature stabilization period. The default pulsing parameters were used for each cycle of TMA (150 sccm carrier gas flow rate; 0.1 s pulse; 3.0 s purge) and H_2_O (200 sccm carrier gas flow rate; 0.1 s pulse; 4.0 s purge). A deposition temperature of 200 °C without further processing was decided upon due to thermal stability considerations for the ITO substrate,^43^ and because these relatively standard conditions are representative of those typically applied in many cases.

Standard samples received 50 cycles of this treatment, for which layer growth was measured to be 1.0 Å/cycle, resulting in layers of Al_2_O_3_ of expected thickness 5.0 nm. A section at the edge of each slide was covered with Kapton tape to remain uninsulated and act as an electrical contact point. ITO/Al_2_O_3_ samples were stored in a sealed container inside the Myfab Uppsala cleanroom environment with a tightly controlled atmospheric humidity and temperature to keep the samples pristine before electrochemical testing.^44^ The modified surfaces were found to be extremely sensitive to mechanical abrasion or disturbance, therefore all samples were maintained face up and handled extremely delicately without touching the conductive side of the ITO.

### 2.3 Electrochemical Measurements

Electrochemical measurements were carried out in a previously reported^45^ custom-designed single-compartment low volume electrochemical cell, adapted to allow careful insertion of the flat working electrodes and direct electronic connection with their uninsulated section, with a modified handmade lid for the counter and reference electrodes. The working electrode was held in place with gentle, evenly dispersed pressure by an in-built screw mechanism against an inert O-ring surrounding the 5 mm diameter circular opening to the solution (Figure S1). In this way, the geometric area exposed was maintained at a constant 0.20 cm^2^ for comparison between samples. All experiments were performed at room temperature with an Ag/AgCl (1.0 M KCl) reference electrode and a graphite rod counter electrode.

Electrochemical solutions were prepared and used fresh. Buffer solutions were made up with 0.1 M total concentration of the buffering species, with the addition of 0.1 M KCl when it was included. pH values were measured using a pH meter and adjusted as necessary with concentrated KOH or HCl, except in the case of chloride-free phosphate buffers, where HCl was avoided. Ferrocenemethanol (FcMeOH) was used as an outer-sphere redox probe at a concentration of 1.0 mM in all solutions, except CV controls for capacitance measurements. 2 mL of fresh solution was carefully pipetted into the cell for each measurement. Application of built-in Ohmic drop compensation by either current interrupt or positive feedback methods was found to have no impact on the voltammograms in this solution for either uninsulated ITO samples or samples modified with 5 or 50 cycles ALD (discussed in Section 3.1).

#### 2.3.1 Electrochemical Impedance Spectroscopy (EIS)

Experiments were performed in the electrochemical cell placed inside a faraday cage. Standard EIS experiments were performed under potentiostatic conditions around 0.190 V with a standard voltage perturbation amplitude of 10 mV in single sine mode. This value was increased to reduce noise for the extremely insulating samples (Table S1). Signal was measured from 10^5^ Hz to 10^–2^ Hz with 10 points per decade for 71 frequencies in total. Measurement time was approximately 15 min per scan. A CV scan under the standard conditions was performed before each EIS measurement.

#### 2.3.2 Cyclic Voltammetry

(CV) was performed on the ITO/5.0 nm Al_2_O_3_ samples, scanning from −0.3 V to +0.7 V vs Ag/AgCl (1.0 M KCl) at a scan rate of 50 mV/s, each cycle lasting 40 s. All voltammetric data is plotted in the IUPAC convention. The first 5 CV cycles were taken at a resolution of 2 mV to check the integrity of the ALD layer and substrate, indicated by a consistent flat shape with currents barely exceeding 100 nA (510 nA/cm^2^) at +0.7 V. Notably higher currents in the shape of sigmoidal microelectrode-like or partial macroelectrode “duck”-like responses indicated initially defective samples, assumed to have preformed pinholes or incomplete or delaminated Al_2_O_3_ films, which was the case for a small fraction of samples, even when no physical cause of damage was apparent. Mishandled samples (e.g., with a scratched surface) or samples which had already been used in the cell and then reinserted would give similar responses. Samples which were fully intact were then tested continuously for a further 3 h 20 min at a lower resolution of 20 mV, with some tested overnight to approach the end state. Experiments were run in at least triplicate to probe the levels of randomness and consistency in behavior between samples, to account for the known variability in properties across regions of ITO glass from the same batch.

## 3 Results and Discussion

### 3.1 Charge Transfer Properties of Samples with Various Barrier Thicknesses

We first investigated the effect of barrier film thickness and aging on charge transfer properties and diffusional processes involving the redox probe. Cyclic voltammetry (CV) and electrochemical impedance spectroscopy (EIS) experiments were performed using samples covered with various thicknesses of Al_2_O_3_ as the working electrodes. Samples with film thicknesses of 5.0, 4.0, 3.0, and 1.6 nm were prepared. As controls, measurements were carried out also for (a) freshly cleaned ITO glass (labeled as “no heat”), (b) ITO glass that was cleaned and then heated up to 200 °C under high vacuum for a few hours under the same conditions as ALD, to simulate any side-effects apart from the vapor deposition (labeled as “vacuum heated”), (c) ITO glass that was exposed to 5 cycles of ALD, too few to form an effective complete layer (labeled as “0.5 nm” for convenience) and (d) ITO glass exposed to 500 cycles of ALD (for 50 nm Al_2_O_3_) as an extremely insulated example.

CVs of oxidation of FcMeOH on electrodes covered with different film thicknesses is shown in Figure 1A. CVs of FcMeOH oxidation at vacuum heated bare electrodes and electrodes covered with 5 cycles of ALD are nearly identical, showing close to reversible behavior with peak separations about 85 mV and estimated charge transfer rate constants of 1.1–1.2 × 10^–2^ cm s^–1^ (calculated from CVs via the Nicholson method,^34,46^ in Supporting Information Section 2.1, Table S2). “No heat” samples meanwhile had a nearly identical response to that of samples modified with a 1.6 nm layer, with peak separation increasing to 120 mV and the charge transfer rate constant decreasing by about three times to 3.6–3.7 × 10^–3^ cm s^–1^. Further increases in film thickness led to larger increases in peak-potential separation and a significant decrease of peak currents. For 3 nm films, this meant the anodic peak shifting by more than 200 mV with the peak currents reduced below half, revealing a peak separation of about half a volt, whereas for 4.0 and 5.0 nm films, this meant the full disappearance of peaks within the default scan range, with a peak separation beyond 1.2 V for 4.0 nm and apparently unascertainable for 5.0 nm (Figure S2A).

**Figure 1:**
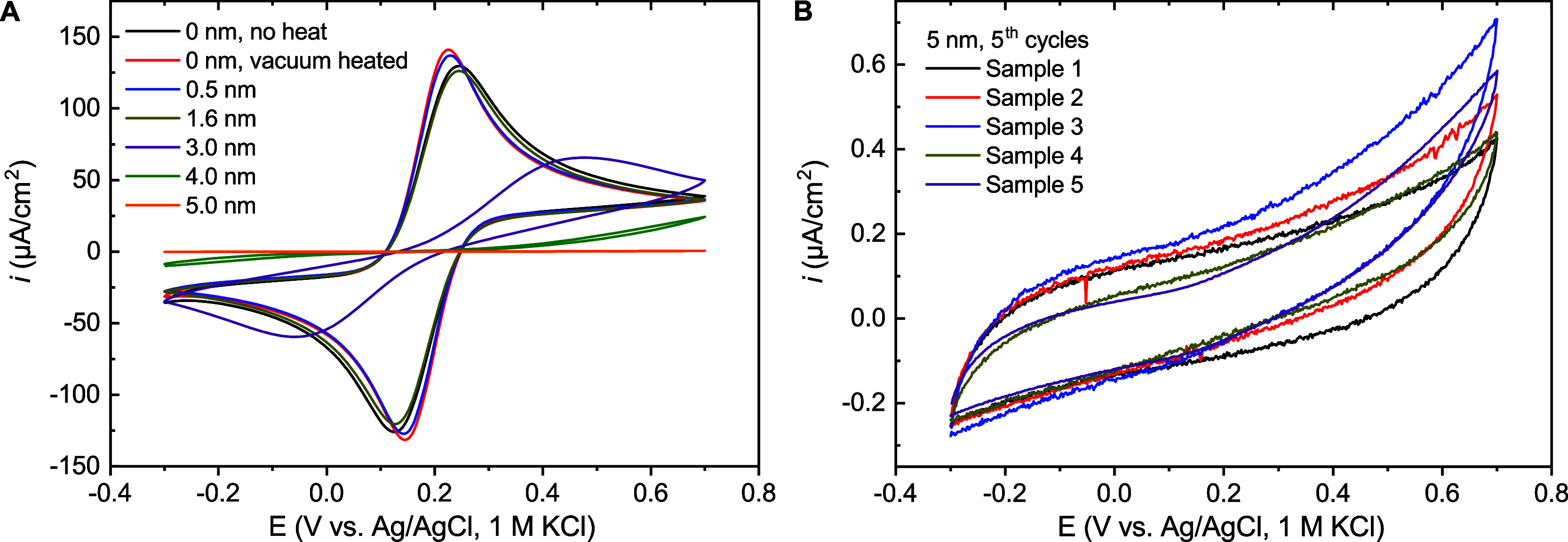
(A) Cyclic voltammograms obtained before impedance measurements for fresh 0.20 cm^2^ samples with different Al_2_O_3_ film thicknesses. Solution: 0.1 M pH 7 potassium phosphate buffer with 1.0 mM FcMeOH. (B) Example cyclic voltammograms obtained for five fresh 0.20 cm^2^ samples with 5 nm Al_2_O_3_ layers (5th cycles shown). Scan rate: 50 mV/s.

Overall, Al_2_O_3_ films of 5.0 nm were found to provide a good level of initial insulation and relative consistency from sample to sample, as shown in Figure 1B. The behavior in these cases differs from the behavior reported for TiO_2_-coated ITO electrodes, where a transition to spherical diffusion was observed for comparable film thicknesses, attributed to pinholes in TiO_2_ layers forming effective ultramicroelectrode arrays.^47^ Tests with 50 nm of insulation layer showed a greater fall in current to below a twentieth of those with 5 nm at +0.7 V vs Ag/AgCl, in this case with a transition in behavior for the remaining current (Figure S2B). Controls for the presence of chloride and the application of different ohmic drop compensation methods were found to have no effect on the behavior observed in CV (Figure S2C–E). Control voltammograms were recorded without FcMeOH in order to observe capacitive currents (Figure S2F).

To further investigate the charge transfer properties of various films, a series of EIS experiments was carried out. A standard Randles circuit with a constant phase element (CPE) and semi-infinite Warburg element (*Z*_W_) for diffusion was used as the default equivalent circuit model for fitting (Scheme 1, EIS fitting details given in Supporting Information Section 2.2). Using this model, values are obtained for the uncompensated resistance accounting for solution resistance, *R*_u_, the specific charge transfer resistance, *R*_ct_, the CPE pseudocapacitance, *Q*_dl_, the CPE exponent, α, and the Warburg coefficient, σ.

**Scheme 1:**
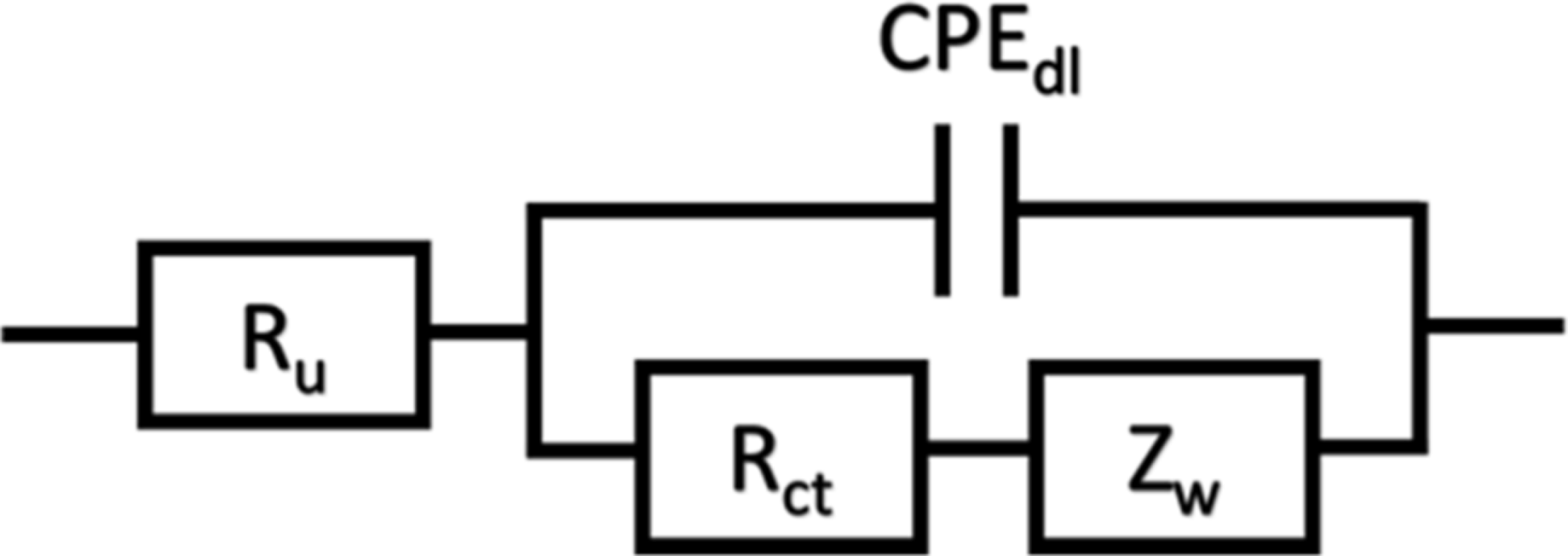
Standard Randles Circuit Used for Fitting EIS Data

Figure 2A,B show Bode plots, with experimental data represented as dots and fits using the equivalent circuit shown in Scheme 1 as lines for all the samples. Heat and vacuum treatment is observed to reduce the charge transfer resistance by over 3 times compared to “no heat’’ samples as was also observed in CV measurements, while also increasing the CPE pseudocapacitance by 6.5 times, although with an associated change of exponent from 0.91 to 0.68. Notably, with 5 cycles of ALD, the reduction in charge transfer resistance again largely remains consistent with CV data, but the CPE pseudocapacitance and exponent return to values similar to those of the unheated glass. These observations may be accounted for by the known effect of heat treatment in promoting rearrangement to remove surface defects and the effect of exposure to single-digit cycles of ALD as a form of surface passivation to remove dangling bonds.^5,6,11,48,49^

**Figure 2:**
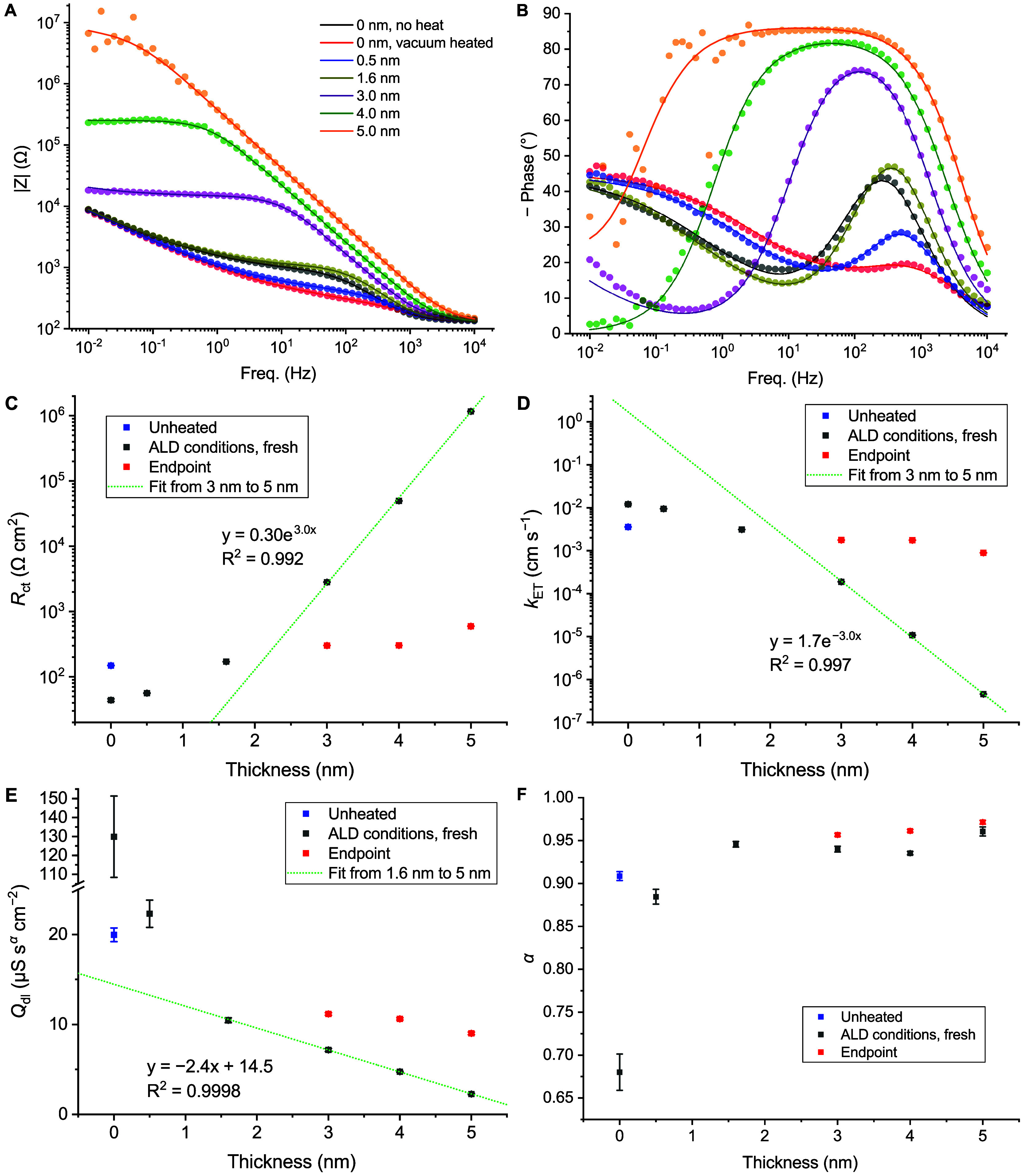
(A) Bode magnitude and (B) Bode phase plots of the fresh 0.20 cm^2^ ITO glass samples in the standard cell setup. Solution: 0.1 M pH 7 potassium phosphate buffer with 1.0 mM FcMeOH. The corresponding Nyquist plots are presented in the Supporting Information (Figure S3). (C–F) The parameters extracted from these EIS experiments for fresh unheated ITO (blue), fresh ALD-exposed samples (gray) and the stabilized end points of 3, 4, and 5 nm samples (red): (C) *R*_ct_, normalized for area and (D) calculated *k*_ET_ values, green lines represent fits applied to 3, 4, 5 nm samples used to extract the tunnelling decay factor. (E) CPE *Q*_dl_, normalized for area, the green line represents the linear fit applied to 1.6–5 nm samples; (F) CPE α. Details for the fitting results and plots of *R*_u_ and σ are given in the Supporting Information (Tables S3–S5 and Figure S4).

ALD of 16 or more cycles had clear effects on the behavior of fresh samples, although these effects diminished with solution exposure time (discussed below). For fresh samples with 1.6 nm, 3.0 nm, 4.0 nm and 5.0 nm thick layers of Al_2_O_3_, the most obvious change is a dramatic increase in charge transfer resistance (Figure 2C), related to the increasing overpotential and interpeak separation seen in the corresponding CVs (Figure 1A). Of note, *R*_ct_ was found to increase exponentially with thickness from 3.0 to 5.0 nm, yielding values for *k*_ET_ which decay exponentially with film thickness (Figure 2D).

At the quantum scale, for individual electrons, direct electron tunnelling through a square energy barrier can be modeled according to^20,21,34,50^

1 $$k_{ET}\left(r\right)=k_{ET}^{{}^{\circ}}e^{-\beta \left(r-r_{0}\right)}$$

where *k*_*ET*_ is the electron transfer rate constant, β is the electron tunnelling decay constant and *r* – *r*_0_ represents the tunnelling distance versus a close-range system, where *r*_0_ is typically taken to be the van der Waals contact distance.^50^ Critically, β depends on the height of the energy barrier and the effective electron mass, and represents how effectively the material hinders tunnelling through itself. Electron tunnelling decay constants for α-Al_2_O_3_ at zero bias have been calculated to be around 0.74–0.78 Å^–1^,^51^ lying between the typical values for aliphatic and aromatic SAMs of 1–1.2 Å^–1^ and 0.4–0.6 Å^–1^, respectively. For reference, this value is estimated to be ∼2 Å^–1^ in vacuum.^20,34^

From fitting points with thickness from 3.0 to 5.0 nm to eq 1, a rough estimate of the tunnelling decay factor β = 0.30 ± 0.01 Å^–1^ was obtained, where *r* is taken to be the tunnelling distance, assumed to be equal to the barrier thickness and *r*_0_ is taken to be 0 as a rough working assumption. This value is significantly lower than the typical values for crystalline Al_2_O_3_ around 0.76 Å^–1^, indicating that the nanoscale amorphous Al_2_O_3_ in our system is behaving as an inferior insulator. This may be due to a combination of the effect of defects in the nanofilm as well as the range of alternative charge transfer mechanisms possible in metal oxides.^1,52,53^

Moreover, at 1.6 nm and below, *k*_ET_ is lower than would be predicted by this trend, showing that other fundamental limits to the electron transfer are at play and reinforcing that the tunnelling barrier is not complete and effective below a certain thickness/number of deposition cycles. Indeed, extrapolation to 0 nm gives an unphysically high rate constant of 1.7 ± 0.5 cm s^–1^. Were the ALD Al_2_O_3_ immediately effective as a tunnelling barrier at any arbitrary thickness, the fall in log­(*k*_ET_) should follow the same steeper gradient already from 0 nm.

One must additionally note the limitations of applying this simple analysis of electron transfer to a complex and amorphous system: For example, any regions of locally thinner Al_2_O_3_ will disproportionately contribute to an increase in the average electron transfer rate for the whole electrode, since the local rate of tunnelling will scale exponentially with the inverse of thickness. The wider the range in Al_2_O_3_ film thickness across the surface, the greater the tunnelling decay factor should be expected to be underestimated. In particular, even very limited numbers of anomalously thin areas, which may be characterized as defects or local breakdowns, would dominate the observed electron transfer behavior. Therefore, extrapolated values of β must be interpreted with caution for these kinds of nonideal macroelectrodes.

Other parameters had notably weaker dependences on thickness; ignoring the exceptionally high CPE pseudocapacitance value for heated ITO, there was a generally mild decrease in double layer pseudocapacitance (Figure 2E), as expected from CVs (Figure 1A) and previous literature.^30,41^ The trend appeared approximately linear from 1.6 to 5 nm, with a gradient of −2.4 μS·s^α^·cm^–2^·nm^–1^, not following the reciprocal relationship observed over this range by Lee et al.^41^ This may be explained by a slower decrease in capacitance with film thickness over this range in our case for reasons related to those discussed in the previous paragraphs, with the system therefore not reaching the same regime.

There was also not a clear significant change in CPE α across this range (Figure 2F). *R*_u_ was not fixed for the fittings, but was found to vary little around 130 Ω, a reasonable value, given the cell geometry and electrolyte concentration. The Warburg coefficient, and therefore extracted diffusion coefficients, likewise tended toward the same final value of 285 Ω·s^–1/2^·cm^2^ across all samples (implying *D* ≈ 7.0 × 10^–6^ cm^2^·s^–1^, in reasonable agreement with the values obtained from the Randles-Ševčík equation from voltammograms of uninsulated samples in Supporting Information Section 2.1) but appeared to be affected to varying degrees by thicker films, and, as discussed before, could not be reliably extracted before the detection of a diffusion-controlled regime at low frequencies, so the values for initial 3.0 nm, 4.0 and 5.0 nm samples are omitted in Figure S4B. All parameters extracted from EIS fitting of various film thicknesses are summarized in Table S6. Finally, controls with a lower FcMeOH concentration of 0.2 mM confirmed the assignments of the circuit elements (Supporting Information Section 2.3, Figure S5).

### 3.2 Stability of Samples with Various Thicknesses in Phosphate Buffer

For the more insulated samples, after the initial CV and EIS characterization, the samples were then cycled under the standard CV conditions 90 times (1 h), before repeating EIS under the same conditions. EIS data was therefore recorded at 75 min intervals to track the progressive changes occurring to these samples (Figure 3). After a number of hours, the CV and EIS responses for each sample stabilized and stopped appreciably changing, and the experiment was halted at 21.25 h, 12.5 h and 5 h for the 5.0, 4.0, and 3.0 nm samples, respectively. The fitted parameters for the final measurement of each are included in Table S7 and (Figure 2C–F) for comparison, and the EIS data of the start and end points is directly compared in Figures S6–S7.

**Figure 3:**
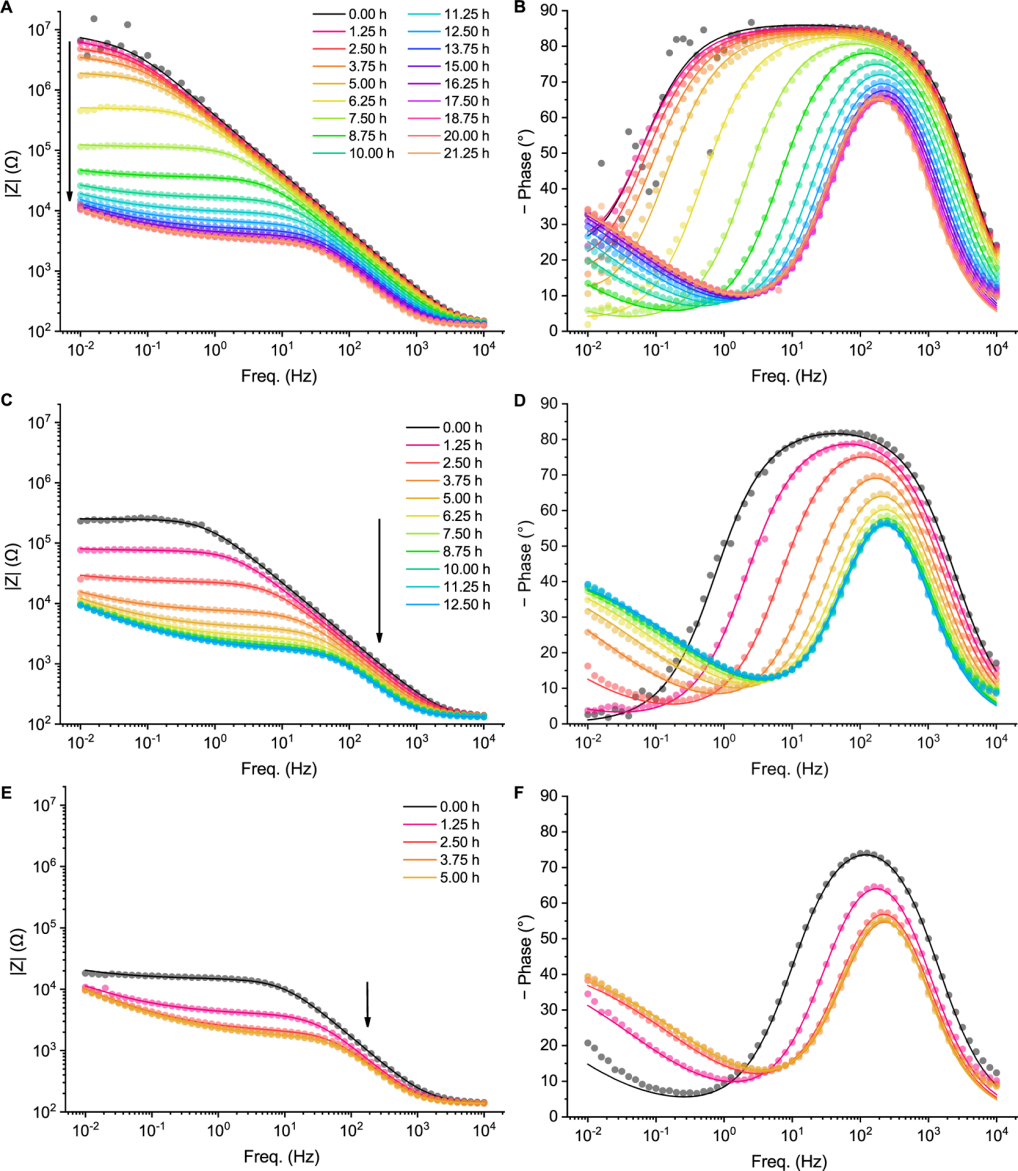
Bode plots over time for 0.20 cm^2^ ITO glass samples with (A,B) 5.0 nm, (C,D) 4.0 nm and (E,F) 3.0 nm Al_2_O_3_ layers showing magnitude (A,C,E) and phase (B,D,E), cycling every 1.25 h until stabilization. Solution: 0.1 M pH 7 potassium phosphate buffer with 1.0 mM FcMeOH. The corresponding Nyquist plots are presented in the Supporting Information (Figures S8–S10).

The layers degrade quite dramatically over several hours in phosphate buffer. Nonetheless, it is notable that the *R*_ct_ values remained higher (and *k*_ET_ remained lower) than for the thinner-film samples even at the end points of these experiments, especially for the 5 nm thick film, while the 3 and 4 nm films’ end points overlap almost completely (Figure 4A), which is especially evident in the Bode and Nyquist plots (Figures S6–S7). This implies that, with sufficient material, some effect from deposition is effectively permanent. One possible explanation is the restructuration of some proportion of the ALD-Al_2_O_3_ into a stable layer of a more resilient polymorph or amorphous phase, depending in part on the solution species present.

**Figure 4:**
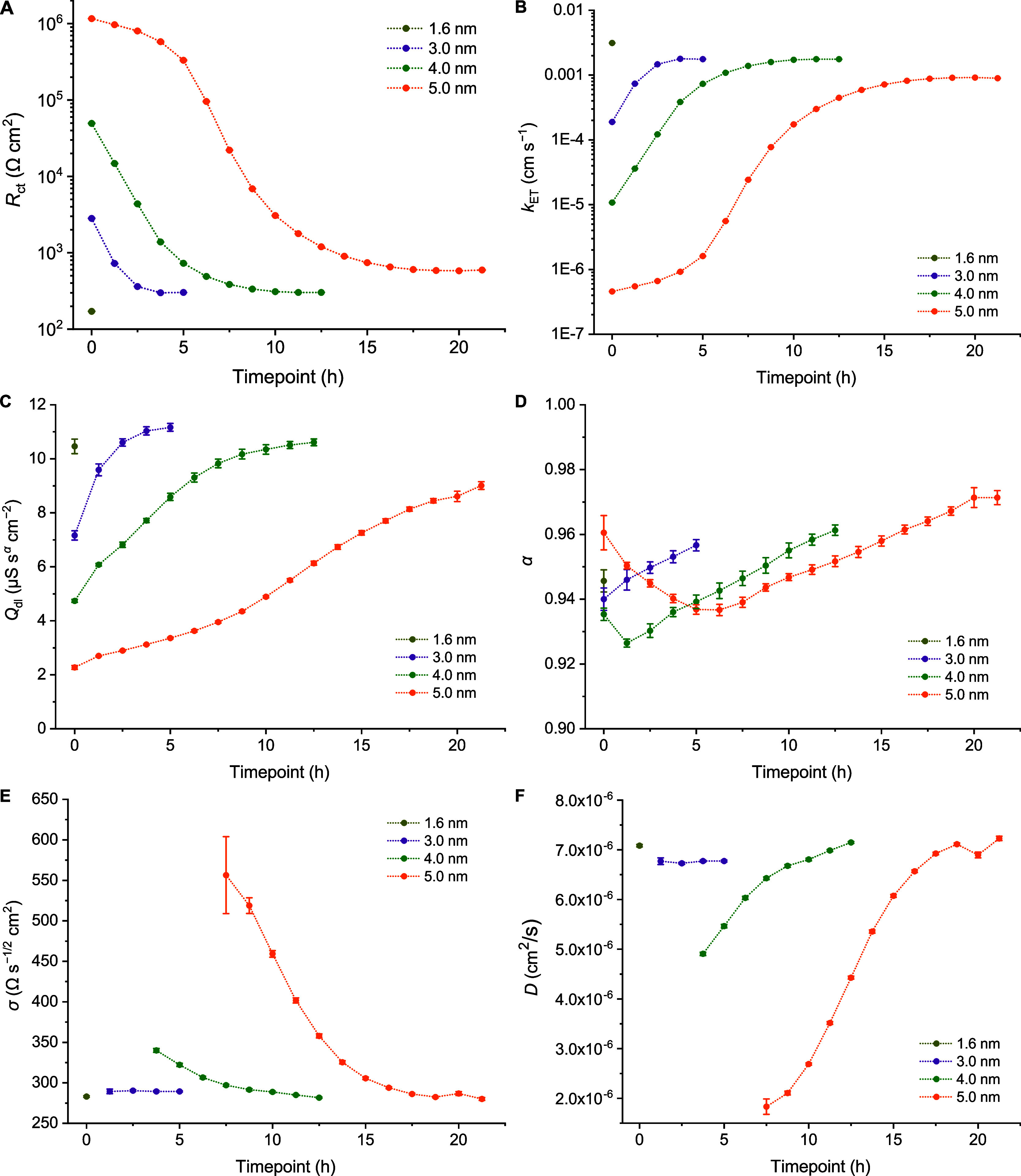
Parameters extracted from the EIS experiments for 0.20 cm^2^ ITO glass samples with 5.0 (orange), 4.0 (green) and 3.0 nm (purple) Al_2_O_3_ layers, cycling every 1.25 h until stabilization. 1.6 nm (gold) at 0 h included for comparison. Solution: 0.1 M pH 7 potassium phosphate buffer with 1.0 mM FcMeOH. (A) *R*_ct_ normalized for area and (B) calculated *k*_ET_ values. (C) CPE *Q*_dl_, normalized for area and (D) CPE α. (E) Warburg coefficients normalized for area and (F) calculated diffusion coefficients. Only values extracted once a diffusion limit was observed are shown. Alternative logarithmic plots with all diffusion data for 5.0 nm are included in the Supporting Information (Figure S11).

Tracking the changes of specific parameters over time, it is apparent that there is a general period of exponential decay of resistance (and associated exponential increase in *k*_ET_), followed by more gradual change until stabilization (Figure 4A,B). With 5.0 nm, however, there is a critical initial period of seemingly linear degradation for the first 5–6 h, which is more rapid in terms of the absolute changes in *R*_ct_, but smaller as a proportion compared to the following exponential period, and thus appears less steep in the first phase of the logarithmic plot. This initial period has a correspondingly very limited growth of *k*_ET_, which would also correspond to the more stable initial phase typically seen for 5.0 nm samples under most conditions in the CV studies (Figure 4B). Altogether, this implies that somewhere between 4 and 5 nm represents the practical lower limit for amorphous Al_2_O_3_ layers on this kind of substrate with these deposition conditions that are reasonably robust to typical buffered aqueous conditions.

Regarding CPE parameters, a continuous increase in pseudocapacitance is observed for all samples, apparently at lower rates for thicker layers (Figure 4C). This is, however, a much less dramatic change than for *R*_ct_, with *Q*_dl_ changing by less than an order of magnitude, even for the 5 nm film over 21 h. Again, this would agree with previous literature, relating a loss of the blocking film to an increased ability to form a double layer.^30,41^ Some change in the process seems to occur around 7.5 h for the 5 nm sample and there does seem to be a tendency toward stabilization with time. Interestingly, the associated α exponents used in fitting seem to dip and then steadily rise again over the course of experiments (Figure 4D). It is unclear if this could be an artifact from equivalent circuit fitting, but, in any case, attributing direct physical meaning to this is fraught with complications.

More intriguingly, the values fitted for the Warburg coefficient (Figure 4E) and the subsequently extracted diffusion coefficients (Figure 4F) display some significant dependence on film age. It is suspected that this implies a thickness dependence, but may also relate to film restructuration, possibly benefiting from increasing unevenness or even some degree of nanoporosity. It is noted that diffusion processes with a 1.6 nm film appear practically indistinguishable from cases with no film (Figures S3–S4 and Table S6), while fresh 3.0 nm appears to be at the borderline of detecting a diffusion limit, so a direct relation of film thickness to the restriction of diffusion for fresh samples cannot be ascertained. Nonetheless, considering some kind of restriction of diffusion appears to be necessary to account for the huge drop-off in peak currents and total charge passed, as well as the observed disappearance of CV peaks altogether (Figure 1A). Albeit, it should be kept in mind that the Warburg element was formulated based on assumptions of semi-infinite planar diffusion,^34,54^ and the values obtained for *D* in these cases can only be specifically representative of the region close to the electrode surface, possibly even entirely within the film layer in some cases, and are therefore not the same as *D* for the redox species in bulk solution.

Although the parameters for diffusion could not be reliably extracted from initial scans for the samples tested, once a layer has degraded enough, electron transfer becomes sufficiently fast that diffusion can become limiting at low frequencies. In the Nyquist plots at different time points (Figures S8–S10), this can be tracked by the appearance of the 45° diagonal line at low frequencies. Like for *Q*_dl_, across the time points where the diffusion limit is clearly observable in Nyquist plots, the change in σ and *D* remains below 1 order of magnitude (Figure 4E,F).

In the case of a 5.0 nm film, algorithmic fitting with the default Randles equivalent circuit was able to extract some rather atypical values for the Warburg coefficient before the 7.5 h mark (Figure 4E), although it is not clear whether there is any real soundness in their derivation.

### 3.3 Stability of Thick Films under Different Electrochemical Experimental Conditions

Given the complexity of the behavior in 0.1 M potassium phosphate buffer at pH 7 even without any other additives, stability tests were carried out over a range of different conditions in order to survey electrochemically relevant buffers and search for possible ways to stabilize the ALD Al_2_O_3_ nanofilms. The survey was restricted to pH 4.0–10.0 using some of the main buffers employed across this range, as previous studies revealed the immediate destructive effect of strong acids and bases.^3,29,42^ Investigation was performed by applying the same standard setup and electrochemical conditions to ITO samples with 5.0 nm ALD Al_2_O_3_ layers in triplicate over 300 CV cycles, corresponding to 3 h 20 min, which should be a reasonable time frame of interest for the fundamental applications discussed in the introduction. A smaller number of samples were left overnight to see if the degradation reached completion in each case over this longer period. An overview of the full set of CV data is given in Figure S12. The degradation of the ALD films in different conditions over time was assessed by following the charge leaked per CV cycle obtained from integration of peaks in CVs, as these values were taken to be the most consistent and directly comparable metric (examples of the obtained CVs and extracted charge leaked per cycle are given in Figure 5). Additional metrics were also evaluated (Figures S13 and S14). From these experiments the effects of individually changing specific conditions could be probed: The effect of pH, the effect of different buffers at the same pH, and the effect of other species in solution, which are discussed below.

**Figure 5:**
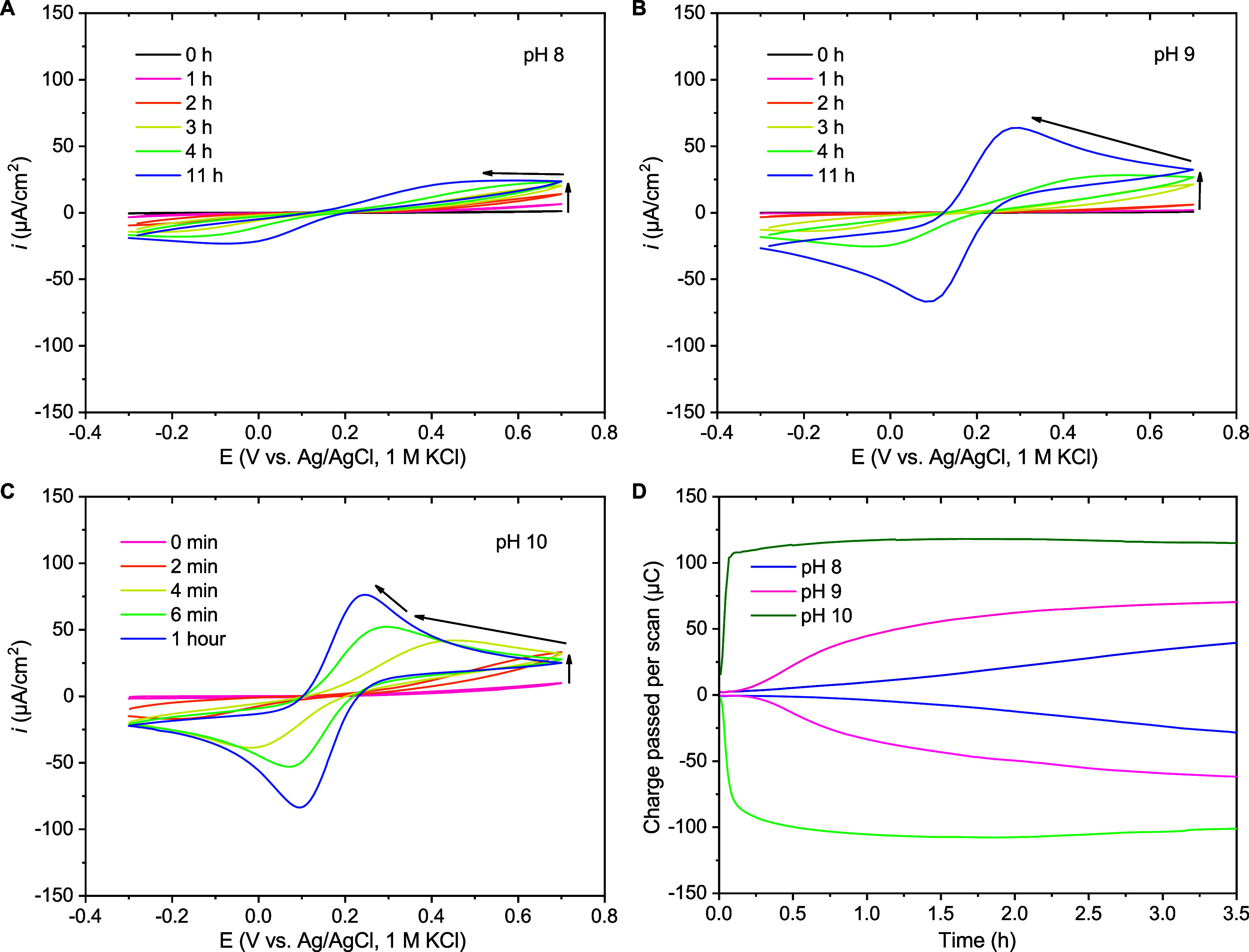
Example voltammograms over time for individual 0.20 cm^2^ ITO samples with 5.0 nm Al_2_O_3_ in 0.1 M borate buffer with 1.0 mM FcMeOH and 0.1 M KCl at (A) pH 8, (B) pH 9, and (C) pH 10. (D) Example plots of the total charge passed per cycle, extracted from the integrals of anodic and cathodic currents for single samples, against time for the first 3.5 h.

### 3.4 Effect of pH

To investigate Al_2_O_3_ insulating layer stability across a wide pH range, the following buffers were made up from their acids and potassium salts at concentrations of 0.1 M with additional 0.1 M KCl as supporting electrolyte: Acetate (pH 4 and pH 5), phosphate (pH 6, 7 and 8), borate (pH 8, 9 and 10). At pHs 8–10 in borate buffer (Figure 6A), the stability of the Al_2_O_3_ layer broadly follows what would be expected from known behavior within the Pourbaix diagram: At pH 10, the system is beyond the expected stable range and consistently dissolves rapidly. At pH 9, there is notably fast degradation on average within half an hour, whereas at pH 8, samples remained mostly intact for over an hour. Broadly speaking, it is therefore observed that moving further from Al_2_O_3_’s optimal stable pH range intuitively accelerates its degradation.

**Figure 6:**
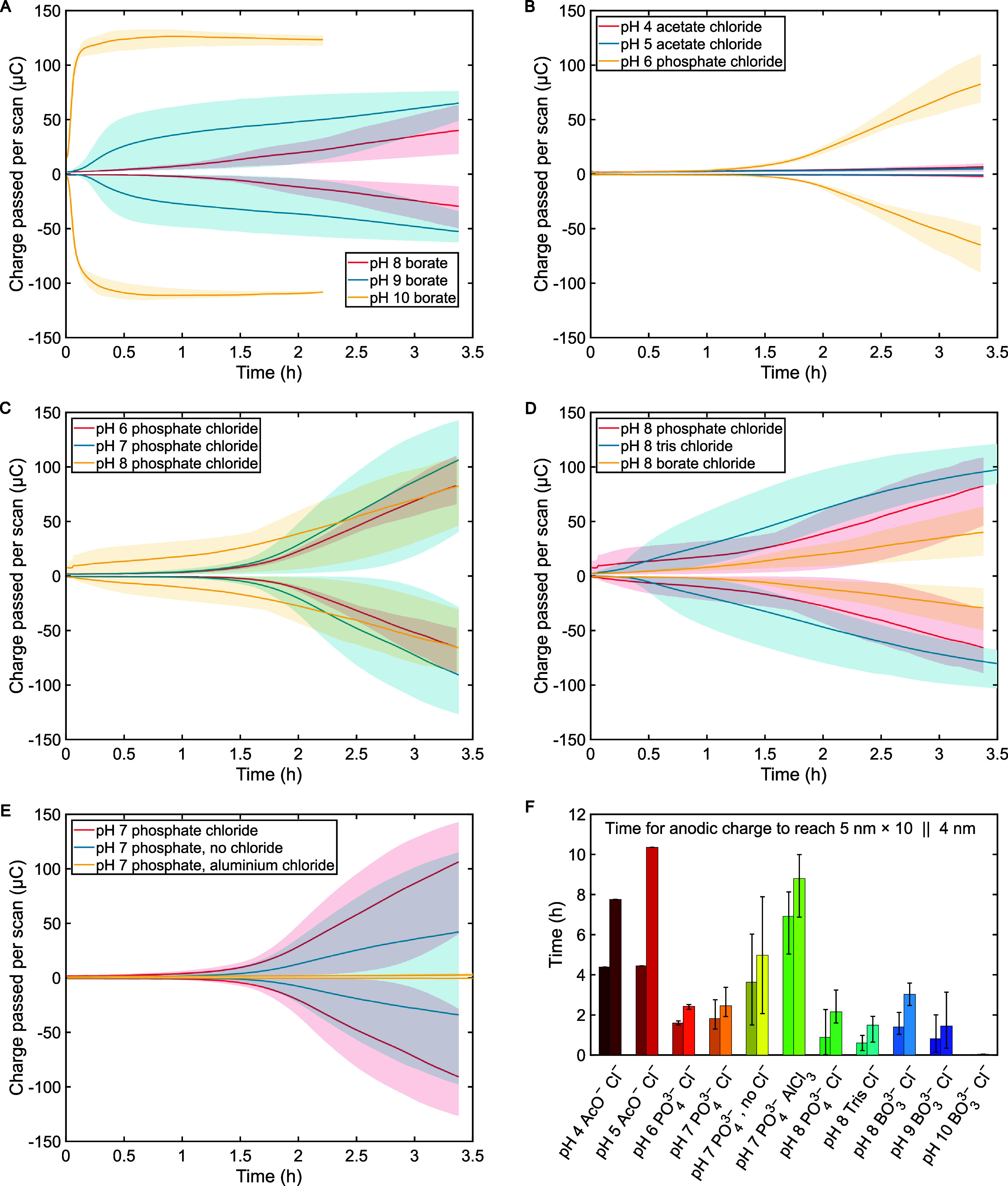
Plots of the total charge passed per cycle, extracted from the integrals of anodic and cathodic currents from CV of each sample, against time for the first 3.5 h. The shaded area represents the range from 3 samples under each condition, with the darker line representing the mean as a guide to the eye. Data sets are selected to directly compare: (A) basic conditions in borate (pH 8, 9, 10); (B) mild acidic conditions (acetate pH 4 and 5 and phosphate pH 6); (C) neutral conditions in phosphate (pH 6, 7, 8); (D) various buffers at pH 8 (phosphate, tris and borate); (E) pH 7 phosphate buffer with 0.1 M KCl, without added chloride, and with 0.33 mM AlCl_3_. (F) Bar charts showing the average time taken for fresh 5 nm samples to reach the point where they leak ten times as much current as fresh 5 nm samples on average (left bars) and as much current as fresh 4 nm samples on average (right bars), based on anodic charge from CV. Ranges across triplicates are shown in error bars, except for acetate conditions, where only one sample in each case was run long enough to reach these points.

Mildly acidic conditions, pH 4–6, correspond to the pH range with the lowest solubility for Al_2_O_3_ and its hydrates.^27^ Despite the intuitive expectation that dissolution would accelerate by at least some degree at pH 4 versus pH 5 as the acidic limit of the possible stable range for Al_2_O_3_ is approached, only a very subtle increase in the average degradation and range was observed when the buffer was acetate in both cases (Figure 6B). In fact, these conditions were found to represent by far the least destabilizing investigated in this study (ignoring controls with added AlCl_3_), evidenced by the almost complete lack of change in leaked current over the first few hours and limited changes even overnight (Figure S12A,B). In stark contrast, despite the small change in pH, degradation in pH 6 phosphate is markedly quicker, completely eclipsing the changes seen in acetate. This is taken to highlight the critical importance of the nature of the solution species, rather than just pH, in terms of the processes at play and the effective layer degradation rate, although the apparently exceptional benignity of acetate is somewhat unanticipated as it has been reported to form complexes with aluminum that are expected to dominate over this pH range.^55^ A further point is that these results seem to generally support the previous observations of slower degradation in acidic conditions than in basic conditions.^29^ At neutral pH values in phosphate buffer a large degree of suddenness in loss of insulation was observed at pH 7 and 8, whereas the behavior of samples in pH 6 was more consistent (Figure 6C).

### 3.5 Effect of Buffer

pH 8 was chosen to test the effect of the buffer as it lies within the range of multiple investigated buffer systems; namely the phosphate, borate and tris buffers (Figure 6D). Although early sudden failure was more significant in phosphate, average degradation in tris overtook the average in phosphate after half an hour, with significant failure observed starting within half an hour to 1 h. This may agree with previous studies by Kim et al. that tris is effectively more destabilizing than phosphate, although the difference is much less marked here.^30^ Borate caused the slowest and least varied degradation behavior and would appear to have been the least problematic at this pH, also reaching lower peak currents than uninsulated ITO controls in neutral conditions. The richness of aqueous borate chemistry and the tendency of borate species to polymerize or bind to other oxides may contribute to some noninnocence in the system,^56,57^ possibly depositing on the surface or otherwise affecting dissolution equilibria (Figure S12I). Although overall the magnitude of current leaking is relatively similar across these buffers, the differences in behavior and variability over time are notable, and this again reinforces the importance of the nature of the species in solution on the performance of the Al_2_O_3_ films.

### 3.6 Effect of Solution Species

To investigate the competing hypotheses on whether the primary culpability lies with chloride or phosphate for failures of Al_2_O_3_ nanofilms in PBS solution, repeat CV controls were also carried out in pH 7 phosphate without chloride. Kim et al. previously compared PBS with a phosphate-free potassium chloride solution, observing a drastically reduced stability without phosphate,^30^ however, without an accompanying buffer for the chloride-only control, it is possible that the bulk solution pH could jump, with repercussions on the Al_2_O_3_ nanofilms, although we also note that local pH at positions on the surface may of course also vary significantly beyond effective control of bulk buffer species. In the present study, the absence of chloride seemed to lower the average leaked current by about half, effectively slowing the rate of degradation by very roughly half on average, but with an increase in the random variation over time (Figure 6E). Although some weak stabilization effect seems to be achieved by removing chloride from the system, the reduced consistency in behavior and the continued appearance of random failure, albeit over a wider timespan, seems to imply that phosphate alone is also sufficiently problematic without chloride for these Al_2_O_3_ films.

Another set of controls was carried out with 0.33 mM AlCl_3_ added to a 0.1 M phosphate buffer solution, which was adjusted to pH 7. Based on the known densities for amorphous Al_2_O_3_ films,^58^ this is estimated to correspond to around 500 times the amount of aluminum in the 5 nm ALD films (Supporting Information Section 2.6). AlCl_3_ was selected as the most convenient soluble source of Al^3+^ in order to control for the presence of Al^3+^ cations in solution, which may shift the chemical equilibria of Al_2_O_3_ against dissolution processes. With the added Al^3+^ ions, there was a very clear effect on the stability of the insulating layer, as the leaked current consistently barely increased over 3 h (Figure 6E). Significant failure of the insulation layer was substantially delayed to between 5–8 h (Figure 6F). It is not clear what true concentration of Al^3+^ was reached in solution or the mechanism of this effect; some kind of reversible restructuration of the Al_2_O_3_ film or a general delaying of the processes behind film breakdown are possible. Nonetheless, this makes for a very interesting result, although the addition of such reactive aluminum salts to many systems may be considered generally impractical as a means of prolonging nanofilm stability.

As a further metric for direct comparison of stability over time, the times taken for the samples to reach certain levels of anodic charge passed per cycle in different buffers were calculated. The levels used for comparison were those for fresh 4 nm samples (to represent the equivalent of losing 1 nm of film) and for fresh 5 nm samples multiplied by 10 (to represent an approximately 90% drop in insulation effectiveness), neglecting a detailed consideration of nonfaradaic contributions to the current. The bar chart in Figure 6F presents the average times these points were reached under each condition, with the error bars representing the range across the triplicates in each case, when available. From this presentation, the superior stability in acetate, the stabilizing effect from added AlCl_3_ salt, and the immediate instability at pH 10 immediately stand out. Overall, we assume the chemical binding of certain solution oxides via reaction with surface hydroxyl groups is possible, while their ability to stabilize Al^3+^ ions in solution or in alternative nonsoluble solid species may also shift the equilibria between possible Al species toward favoring degradation of Al_2_O_3_. Presumably, the presence of Al^3+^ already in solution has the opposite effect. The role of chloride remains unclear, although halides are known to effectively penetrate Al_2_O_3_.^27,35,36,38^ In the case of multiprotic acids, bound conjugate base species may play a role as proton relays and in influencing local pH, which may expedite degradation processes kinetically. We note that acetic acid was both the least problematic buffer and the only monoprotic acid investigated (meaning it would be unable to act as a proton relay in the same way as e.g. phosphate), although degradation behavior under acidic conditions is of course different due to the different equilibria at play.

It was generally noted that the loss of insulation could appear and accelerate suddenly, tentatively suspected to correspond to regional breakdown of the films, giving the degradation process a degree of randomness and unpredictability. This appeared to be far more pronounced in the mildly basic conditions (pH 7–9) investigated, where AlO_2_^–^ would be the dominant oxide solution species and a basic dissolution mechanism could be expected to predominate. There may be a number of physical contributing factors: Defects or inhomogeneities in the commercial substrate, tiny flaws in sample treatment and ALD preparation from dust or minor abrasions, or mechanical stress from handling and insertion into the electrochemical cell. Both kinds of processes may be at play in the same system: Sudden, unrelated failures may happen in cases that have predictable gradual dissolution initially. An initial local breakdown in one area may accelerate breakdown in the surrounding area due to the increased current passing locally and its consequences – breakdowns might be effectively autocatalytic.

### 3.7 EIS vs CV Comparison with Fitting and Discussion of Degradation Phenomena

In order to gain insight regarding the physical factors behind the changing voltammogram profiles observed over the course of the stability studies (Figure S12) and the processes involved in the loss of insulation from Al_2_O_3_ films, the key parameters extracted from EIS for a 5 nm film over time were used to produce simulated voltammograms, to compare directly with the CVs recorded directly before each EIS scan (Figure 1A). Homemade CV simulation code from our group based on diffusional modeling and the Butler–Volmer equation was used.^59^ Full details on specific parameters, assumptions and boundary conditions are given in ref 60 (Chapter 7). The standard CV conditions were inputted into the code, along with the relevant concentrations, measured *E*_1/2_, and values for *D* and *k*_ET_ extracted from EIS measurements of the 5 nm sample at the time points over the course of the experiment (shown in Figure 4). This model was validated for the uninsulated samples (Figure S15).

Using the calculated values of *D* and *k*_ET_ for each scan allowed the magnitude of the current to be reproduced with reasonable fidelity over the whole time scale (Figure 7A vs B), even for the points which used diffusion coefficients extracted before 7.5 h of cycling, something which is not realized when diffusion is neglected (Figure S16). Nonetheless, the profiles of these voltammograms still incorrectly predict much sharper peaks than are actually observed, particularly at the earliest time points, when the real data did not reach peaks in either scan direction across the standard scan range (Figure 7C vs D). Furthermore, the peak separations in the simulated voltammograms are incongruously lower at the start than at the intermediate time points, with the peak potentials gradually diverging, before coming back together. The same simulation was applied to the 4 nm data (Figure S17) and the concentration controls with 0.2 mM FcMeOH (Figure S18).

**Figure 7:**
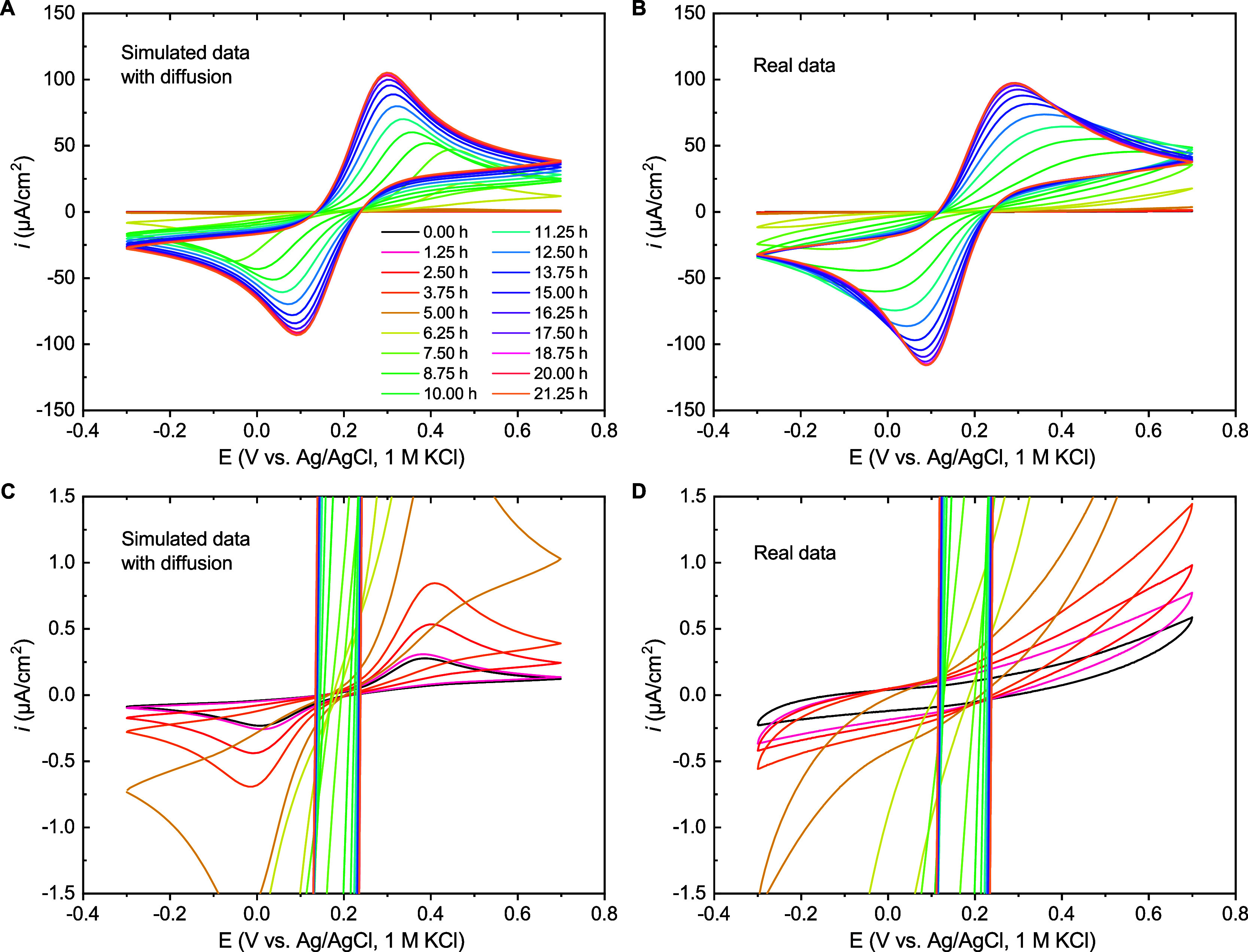
(A) Simulated CV data based on *k*_ET_ and *D* values extracted from EIS versus (B) the real CV data recorded before each EIS scan for the 0.20 cm^2^ ITO sample with 5.0 nm Al_2_O_3_ over the EIS measurement time points until stabilization. Solution: 0.1 M pH 7 potassium phosphate buffer with 1.0 mM FcMeOH. (C,D) Zoomed 100× into low currents to highlight the profiles of the early scans.

Although it remains unclear exactly how the largely featureless profile of the early real 5.0 nm data can be mathematically modeled precisely, it appears that the model applied here is somewhat too naïve and proceeds to predict that a diffusional limit will still be reached within our standard CV potential range due to the Butler–Volmer equation’s assumptions. Cursory tests using the classic Marcus–Hush model with typical values for reorganization energy did not produce substantially different simulated voltammograms. The more sophisticated Marcus–Hush–Chidsey model is known to account for peak broadening in certain situations.^61,62^ However, due to the extreme broadening observed and the lack of a clear physical reason to expect significant changes in reorganization energy, we hypothesize that a distribution of *k*_ET_ arising from some variation in local film thickness is the main cause for the peak broadening.^61^

Altogether, the complexity of such systems is nontrivial, particularly in cases with formation of nanopores or pinholes;^47^ certain assumptions based on more typical conditions must break down at this point. Regardless, incorporating hindered diffusion at the surface into the model appears to be a step forward in understanding the underlying processes by which Al_2_O_3_ insulates the surface to redox active solution species. It is unclear whether the higher values of *D* ultimately represent FcMeOH having to partially penetrate the outer film in order to reduce the tunnelling distance, somewhat akin to the process by which halides penetrate Al_2_O_3_ to corrode aluminum metal,^27,35,36,38^ or whether it may represent some diffusion of charge through the Al_2_O_3_ in the form of ion mobility and local restructuring of the metal oxide, a process which would also be critically distance-dependent. The greater size of the FcMeOH molecule compared with Cl^–^, its ability to adsorb via intermolecular forces with its polar groups, and its change from neutral charge to positive charge upon oxidation must all be considered.

## 4 Conclusions

We have presented a systematic study into the performance of nanoscale ALD–Al_2_O_3_ layers on conductive indium tin oxide (ITO) glass as tunnelling barriers for (photo)­electrochemical systems alongside a methodology to assess their stability over time using ferrocenemethanol as a redox probe in solution. From this, we highlight the critical importance of a number of factors in the application of these layers. First, deposition of a certain thickness is required to reach a point of greater stability in solution. For this system, this was observed to be between 4 and 5 nm. This is distinct and significantly more than the lowest number of deposition cycles required to achieve a continuous film. Second, the specific ions in solution play a major role in the effective lifetime of the insulating layer. Many common buffers are found to be detrimental, including phosphate, while chloride does not seem to be problematic *per se*, at least in mild acidic conditions, but may play some role in accelerating degradation alongside other species. Relatively low concentrations of the aluminum ions seem to have a significant stabilizing effect, delaying breakdown by multiple hours. Meanwhile, pH was only found to play a significant direct role below pH 4 or above pH 8. Notably, acetate was significantly less destabilizing than other buffers tested, although the specific mechanistic reasons remain unelucidated. Third, there is an element of randomness in the loss of insulation across samples, which often starts suddenly with some apparent degree of unpredictability even under the same conditions, but the voltammetric profiles do not appear to support extremely localized failure at few, specific, isolated pinholes. Dealing rigorously with variation in performance across samples with ALD thin films represents a challenge for the field and this reinforces the importance of repeat experiments to gain statistical information.

In terms of the electrochemical behavior, a certain thickness has to be reached before the electron transfer rate through the film decays with distance according to the exponential relationship expected by a simple tunnelling model. The extracted decay constants in this system were notably lower than literature values for crystalline Al_2_O_3_. Similarly, the decrease in double layer capacitance per film thickness was lower than expected and the expected reciprocal relationship^41^ was not reached. Additionally, diffusion coefficients extracted from EIS seem to indicate an effective reduction in the mobility of the redox probe molecules for films above 3 nm in thickness, implying that these films effectively play multiple roles in hindering electron transfer to dissolved species. That is, it is possible that a slight penetration of the outer parts of the films results in a region of slower charge diffusion near the electrode, which may be detected because it achieves a reduced electron tunnelling distance at points where the charge transfer resistance is extremely limiting so that small reductions in tunnelling distance (by e.g. FcMeOH partially penetrating the film) speed up charge transfer significantly. Taking all these parameters into account with sufficiently sophisticated models is necessary to accurately predict the observed current responses.

Considering that the as-deposited Al_2_O_3_ films were amorphous by nature, their kinetic instability is the major issue in their practical application. Some possible strategies exist to mitigate this: If compatible with the substrate, samples can be treated with ozone/plasma/higher temperatures during ALD or annealed afterwards to improve crystallinity.^3,31–33^ Otherwise, selecting a substrate with improved nucleation behavior for TMA/H_2_O ALD or minimizing the roughness of the substrate may increase ALD film crystallinity.^26^ Alternative wide-bandgap insulators (e.g., HfO_2_, Ta_2_O_5_, SiO_2_) may be viable for compatible systems,^2^ while mixed ALD layers are also reported to have some improved properties.^63,64^ Once exposed to solution, however, insulation appears only to decrease and this can only be slowed, not reversed. Nonetheless, under the conditions investigated here with quite standard ALD conditions on a typical conductive glass substrate, 5 nm films of Al_2_O_3_ reliably provided a consistent insulating effect for more than an hour over the theoretical stable pH range, in some cases lasting several hours.

## Supplementary Material



## Notes

Experimental details; calculation of charge transfer constants and diffusion coefficients from CV data; control CVs; additional EIS data; details of EIS fitting; effect of FcMeOH concentration on EIS; additional CV data for stability testing; and details of CV simulations (PDF)
