# Supplementary material for: Re-Evaluating the Stability of Al2O3 Barriers Prepared by Atomic Layer Deposition under Electrochemical Conditions
Source: ACS Appl Mater Interfaces. 2025 Aug 19;17(34):48320–33. doi: 10.1021/acsami.5c11388 (PMC12400279; doi:10.1021/acsami.5c11388)
Supplement: Supplementary file 1 [file am5c11388_si_001.pdf]

Supporting Information

Re-evaluating the Stability of Al_2O_3 Barriers Prepared by Atomic Layer Deposition under Electrochemical Conditions

*Andrew J. Bagnall, Ziwen Zhao, Mun Hon Cheah, Alina Sekretareva**

Department of Chemistry – Ångström Laboratory, Uppsala University, 75120 Uppsala (Sweden)

E-mail: alina.sekretareva@kemi.uu.se

Table of Contents

Table of Contents 2

1. Experimental Details 4

Figure S1. 5

2. Results and Discussion 6

2.1. Charge transfer properties versus barrier thickness in CV 6

Figure S2. 7

2.2. EIS Fitting Details and Charge Transfer Properties versus Barrier Thickness 8

Figure S3. 9

Figure S4. 11

Table S6. Fitted Equivalent Circuit Model Parameters for ITO Samples. 11

2.3. Effect of FcMeOH Concentration on EIS Responses 12

Figure S5. 12

2.4. EIS Comparisons of 5.0 nm, 4.0 nm and 3.0 nm Insulated Samples over Time 13

Figure S6. 13

Figure S7. 13

Figure S8. 14

Figure S9. 15

Figure S10. 15

Figure S11. 16

Table S7. Fitted Equivalent Circuit Model Parameters for 3 nm, 4 nm and 5 nm Samples at Regular Timepoints over Cycling. 17

2.5. Original CV Data Used for Stability Assessments in Different Solutions 18

Figure S12. 21

Figure S13. 22

Figure S14. 23

2.6. Estimate of Moles of Aluminium Deposited 24

2.7. Further Details on CV Simulations Based on Parameters Extracted from EIS 25

Figure S15. 25

Figure S16. 25

Figure S17. 26

3. References 27

1. Experimental Details

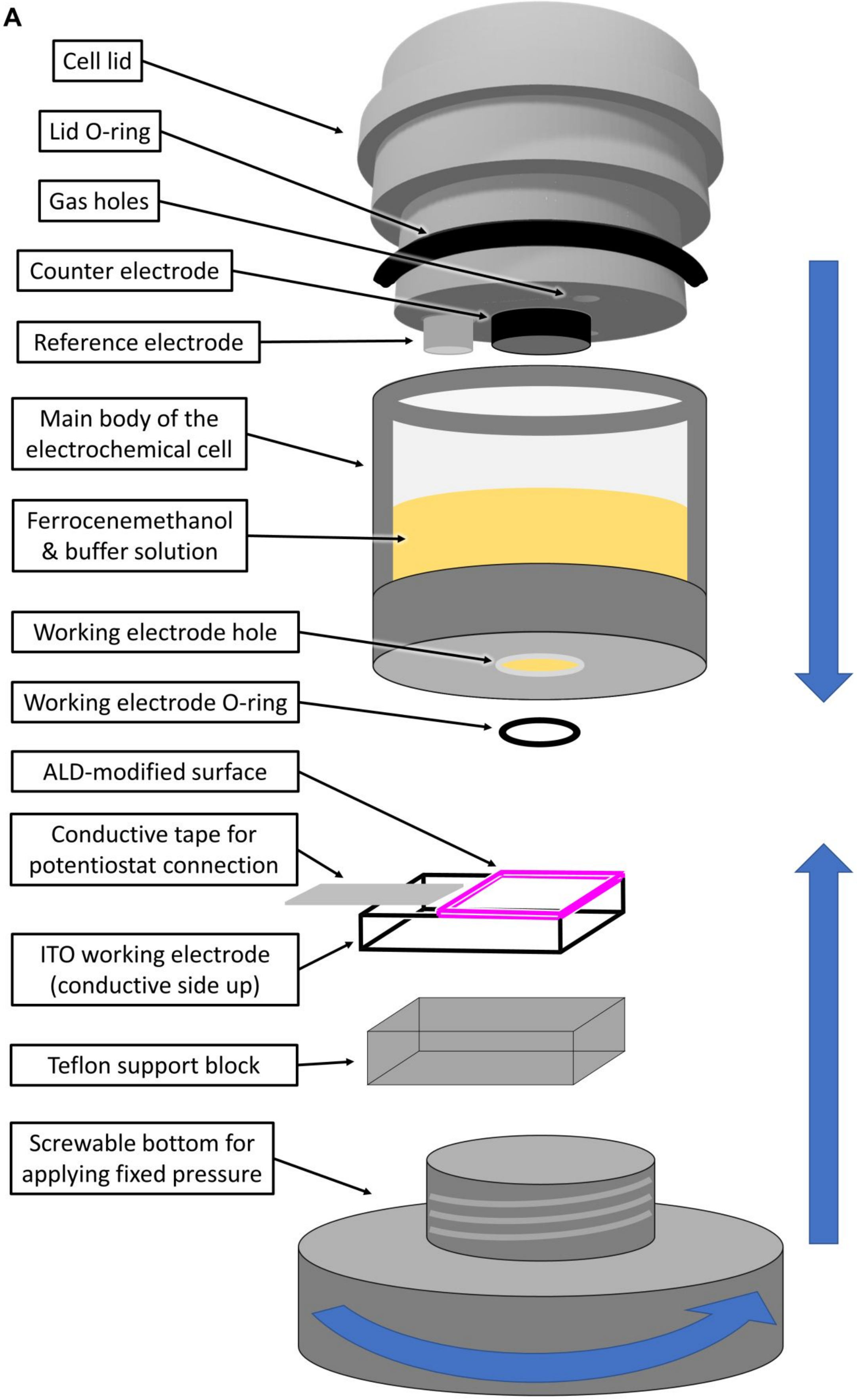

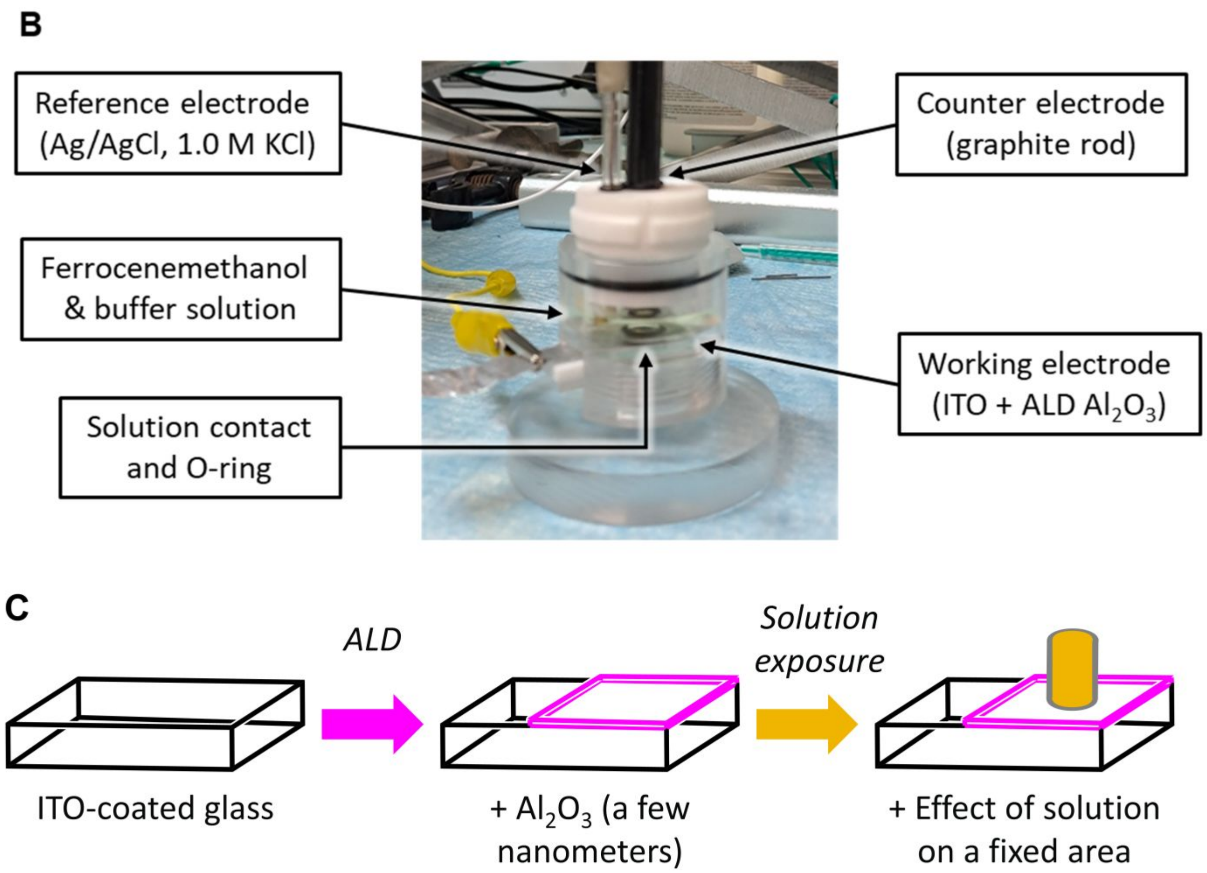

Figure S1. (A) Schematic illustration of the custom-designed electrochemical cell and its assembly. Detailed depiction of the cell main body's screw mechanism and groove for holding the working electrode in place is omitted for simplicity. Components are not represented to scale. Note that the solution and lid are inserted *after* the working electrode is screwed in place. (B) Photograph of the electrochemical cell setup in operation. (C) Simplified illustration of the ALD-modified working electrodes and their electrochemically studied exposed area, reliably controlled by this setup to be 0.20 cm^2.

Table S1. EIS Voltage Perturbations Applied by Experiment.

Sample	EIS Scan no.	Timepoints (h)	Voltage Perturbation (mV)
ITO/3 nm Al_2O_3	1	0	20
	2–5	1.25–5	10
ITO/4 nm Al_2O_3	1	0	50
	2–11	1.25–12.5	10
ITO/5 nm Al_2O_3	1	0	100
	2–3	1.25–2.5	50
	4–5	3.75–5	20
	6–18	6.25–21.25	10
All other samples	-	-	10

2. Results and Discussion

2.1. Charge transfer properties versus barrier thickness in CV

Extraction of Charge Transfer Rate Constants from CV

Heterogeneous charge transfer rate constants were extracted from the voltammograms in Figure 1 from the peak separations using the Nicholson method[1,2] with diffusion coefficients estimated from the anodic peaks via the Randles-Ševčík equation:

$$i_p = 0.4463\, nFAC\left(\frac{nFvD}{RT}\right)^{\frac{1}{2}} \qquad \text{(S1)}$$

Where n is the number of electrons, F is the Faraday constant, A is the electrode surface area, C is the concentration of the redox probe, v is the scan rate, D is the diffusion coefficient (assumed to be the same for the oxidised and reduced forms of the redox probe), R is the ideal gas constant and T is the absolute temperature.

$$k_{\mathrm{ET}} = \Psi\left(\pi D\,\frac{nFv}{RT}\right)^{\frac{1}{2}} \qquad \text{(S2)}$$

Where Ψ is a dimensionless parameter related to the electrochemical reversibility of the system, determined by the peak separation.

Table S2. Parameters estimated from CV controls.

Sample	i_{pa} (µA/cm^2)	D (cm^2/s)	ΔE (mV)	Ψ	k_{ET} (cm/s)
ITO/0 nm heated	141	5.51E-06	~85	1	5.81E-03
ITO/0.5 nm	137	5.19E-06	~85	1	5.64E-03
ITO/0 nm no heat	130	4.67E-06	~120	0.35	1.87E-03
ITO/1.6 nm	126	4.42E-06	~120	0.35	1.82E-03
ITO/3.0 nm	66	1.20E-06	~500	*n/a*	*n/a*

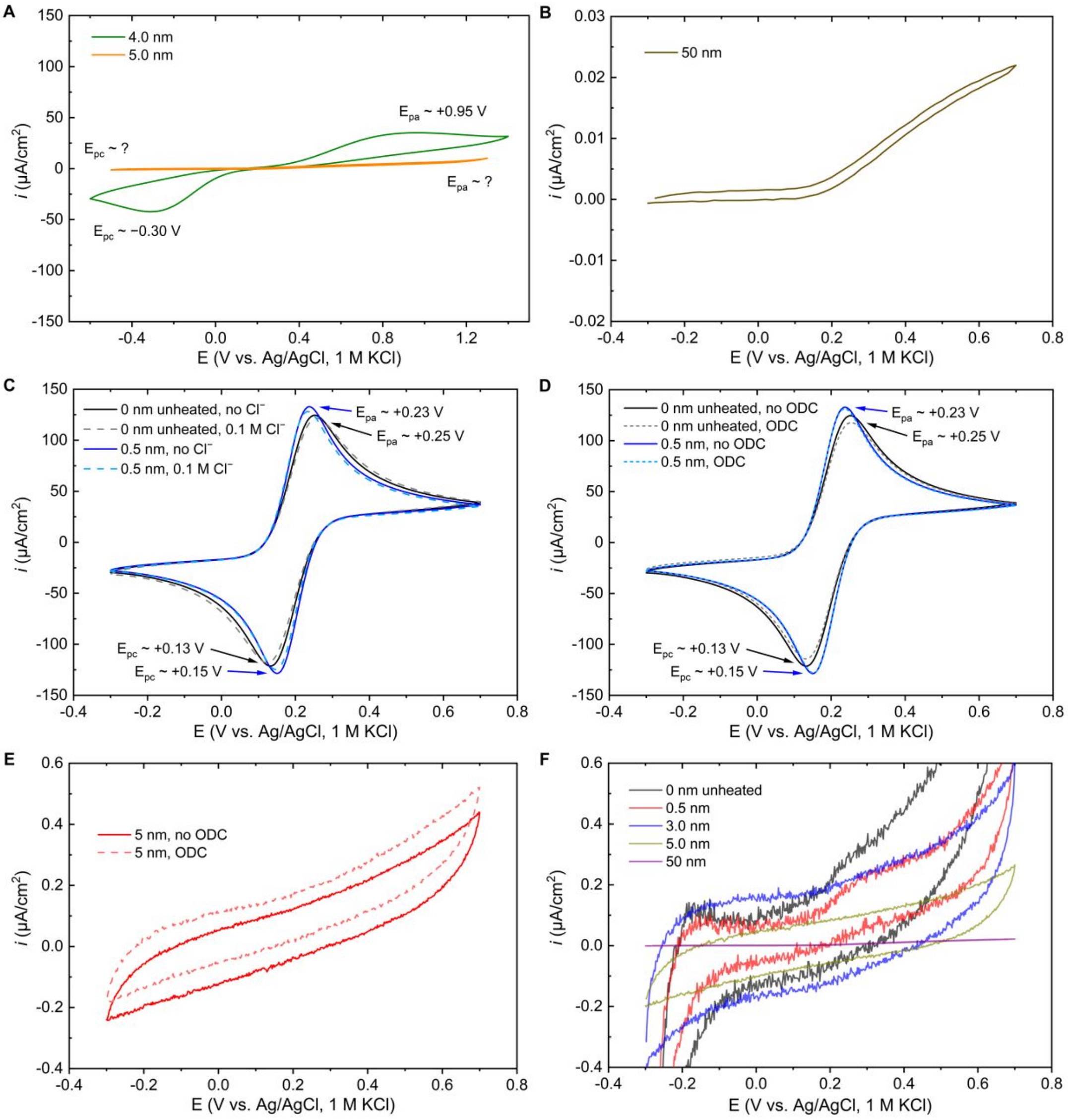

Figure S2. CV control experiments. 5th cycles shown. Solution: pH 7 0.1 M phosphate buffer, with 1 mM FcMeOH (A–E). (A) 4 nm (green) and 5 nm (orange) samples scanned over a wider range. (B) 50 nm sample scanned over the standard range, with 0.1 M KCl. (C) CVs of unheated ITO (black) and samples treated with 5 cycles Al_2O_3 ALD (blue) without chloride (solid lines) and with 0.1 M KCl (dashed lines), showing no significant changes. (D–E) CVs without chloride of the same samples and a sample with 50 cycles ALD (red) without ohmic drop compensation (solid lines) and repeats with ohmic drop compensation (positive feedback method, short dashed lines). The current interrupt method had an identical effect to positive feedback, again, showing no significant changes. (F) Control CVs without FcMeOH for selected samples, showing too much deviation in current to extract comparable capacitance values (recorded with 0.1 M KCl; CVs recorded without chloride were very similar).

2.2. EIS Fitting Details and Charge Transfer Properties versus Barrier Thickness

For fitting the recorded EIS data using the Randles circuit (Scheme 1), a CPE instead of an ideal capacitor was necessary to fit the Nyquist plot arcs well, with exponent values generally within the range 0.92–0.97. In all cases tested, this model proved to provide the most satisfactory fit, noting that the Warburg element could be neglected when diffusion could not be observed at low frequencies as a 45° diagonal line. Alternative typical equivalent circuits derived for failed film coatings[3] or incorporating a Young impedance to model an uneven distribution of resistivity through the film[4,5] were tested but were not found to provide superior fitting.

Data fitting was carried out using the impedance.py Python package,[6] with modifications to include the Young impedance as a custom element and iteratively test various possible equivalent circuits across all datasets.

From R_{ct}, the electron transfer rate constant, k_{ET}, can be calculated by Equation S3:[1]

$$R_{ct} = \frac{RT}{F^2 k^0_{ET} C} \qquad \text{(S3)}$$

where R_{ct} is normalised to area and in units of $\Omega{\cdot}cm^2$ and C is taken to be half the initial concentration of FcMeOH, as it is estimated that half of it is oxidised to $FcMeOH^+$ from the continuous application of its $E_{1/2}$. A single-electron process is assumed. From σ, the diffusion coefficient, D, assumed again to be the same for both FcMeOH and $FcMeOH^+$, can be calculated by Equation S4:[1]

$$\sigma = \frac{2RT}{F^2 \sqrt{2} \sqrt{D}\, C} \qquad \text{(S4)}$$

where σ is likewise normalised to area and in units of $\Omega{\cdot}s^{-1/2}{\cdot}cm^2$.

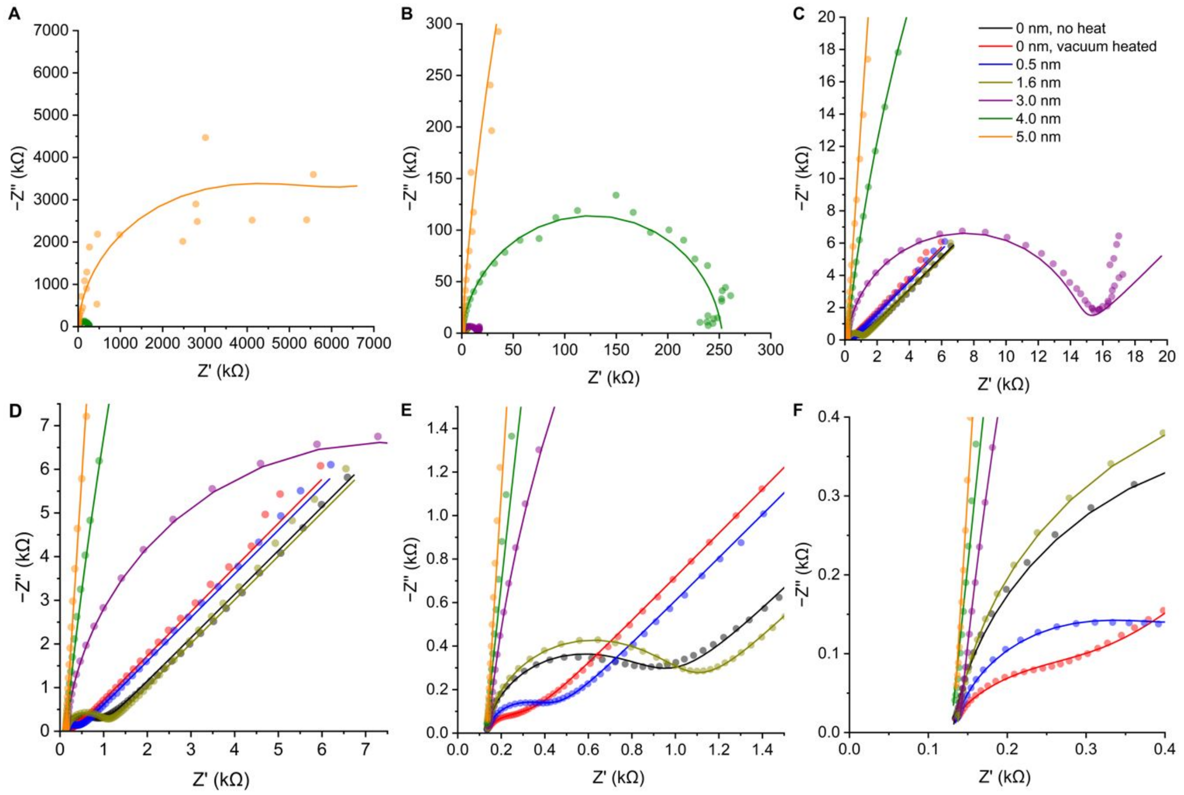

Figure S3. Initial Nyquist plots of fresh 0.20 cm^2 ITO glass samples in the standard cell setup. The same data is presented with axes scaled to (A) 7000, (B) 300, (C) 20, (D) 7.5, (E) 1.5 and (F) 0.4 kΩ. Solution: 0.1 M pH 7 potassium phosphate buffer with 1.0 mM FcMeOH.

Table S3. Fitting RCT vs. film thickness for Figure 2C.

Model	ExpGro1		y = R_{ct}	
Equation	y = A1*exp(x/t1) + y0			
Plot	A.R1_ct			
y0	0 ± 0			
A1	0.29948	±	0.0917	/Ω cm^2
t1	0.32982	±	0.0073	/nm
	3.29820	±	0.0732	/Å
**1/t1** ***(growth factor)***	**0.303196**	**±**	**0.0067**	**/$Å^{-1}$**
Reduced Chi-Sqr	237.8785			
R-Square (COD)	0.99665			
Adj. R-Square	0.9933			

Table S4. Fitting kET vs. film thickness for Figure 2D.

Model	ExpDec1		y = k_{ET}	
Equation	y = A1*exp(−x/t1) + y0			
Plot	k_ET_0			
y0	0 ± 0			
A1	1.71044	±	0.4555	/cm s^{-1}
t1	0.33059	±	0.0064	/nm
	3.30590	±	0.0639	/Å
**1/t1** ***(decay factor)***	**0.30249**	**±**	**0.0058**	**/$Å^{-1}$**
Reduced Chi-Sqr	200.169			
R-Square (COD)	0.99298			
Adj. R-Square	0.98596			

Table S5. Fitting CPE Q_{dl} vs. film thickness for Figure 2E.

Model	LinearFit		y = Q_{dl}	
Equation	y = a + b*x			
Plot	Q_dl			
Weight	Instrumental			
Intercept	14.46291	±	0.09332	$\mu S \cdot s^{\alpha} \cdot cm^{-2}$
**Slope**	**−2.43514**	**±**	**0.02173**	**$\mu S \cdot s^{\alpha} \cdot cm^{-2} \cdot nm^{-1}$**
Residual Sum of Squares	0.26597			
Pearson's r	-0.99992			
R-Square (COD)	0.99984			
Adj. R-Square	0.99976			

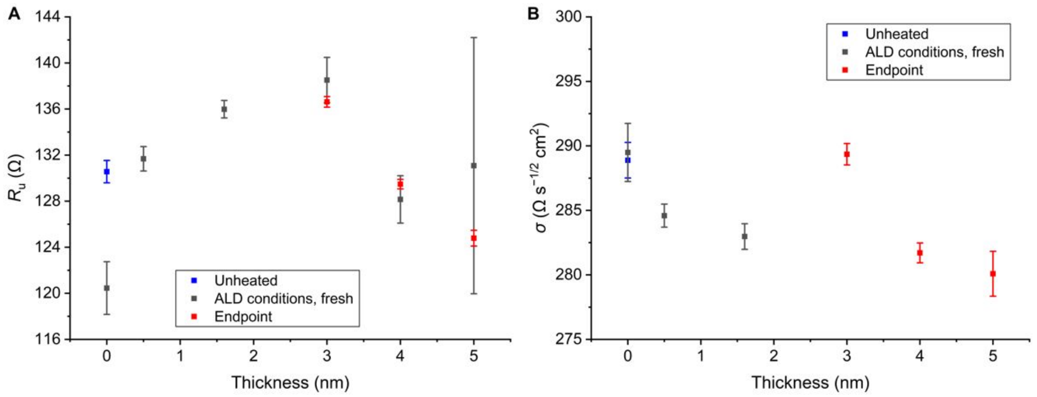

Figure S4. (A) Uncompensated (solution) resistances obtained from EIS fitting, not adjusted for area. (B) Warburg coefficients, adjusted for area, except for fresh samples with ≥ 3.0 nm, where the diffusion was not directly observable due to the lack of the 45° diagonal at low frequencies in the Nyquist plots. Note the closeness of values across all samples in these cases.

Table S6. Fitted Equivalent Circuit Model Parameters for ITO Samples.

Sample	R_u (Ω·cm^2)	R_{ct} (Ω·cm^2)	Q_{dl} (μS·s$^{\alpha}$·cm^{-2})	a	σ (Ω·s$^{-1/2}$·cm^2)	k_{ET} (cm·s^{-1})	D (cm^2·s^{-1})
0 nm unheated	26	150	20	0.91	290	3.6×10^{-3}	6.8×10^{-6}
0 nm heated	24	44	130	0.68	290	1.2×10^{-2}	6.8×10^{-6}
0.5 nm	26	56	22	0.89	280	9.5×10^{-3}	7.0×10^{-6}
1.6 nm	27	170	10	0.95	280	3.1×10^{-3}	7.1×10^{-6}
3 nm initial	27	2.8×10^3	7.2	0.94	250 *	1.9×10^{-4}	8.8×10^{-6} *
4 nm initial	25	5.0×10^4	4.7	0.94	19 *	1.1×10^{-5}	1.6×10^{-3} *
5 nm initial	26	1.2×10^6	2.3	0.96	9.7×10^4 *	4.6×10^{-7}	6.0×10^{-11} *
3 nm end	27	300	11	0.96	290	1.8×10^{-3}	6.8×10^{-6}
4 nm end	25	300	11	0.96	280	1.8×10^{-3}	7.1×10^{-6}
5 nm end	25	600	9.0	0.97	280	8.9×10^{-4}	7.2×10^{-6}

* Values extracted before reliable observation of diffusion.

2.3. Effect of FcMeOH Concentration on EIS Responses

Controls with 0.2 mM FcMeOH vs. 1.0 mM FcMeOH

The identification of the main arc in the Nyquist plots as relating to the charge transfer resistance for reduction/oxidation of ferrocenemethanol and the diffusional diagonal line as relating primarily to the diffusion of ferrocenemethanol was confirmed by concentration controls. For certain samples, at the end of the main experiment, the buffer solution containing 1.0 mM ferrocenemethanol was replaced with an otherwise identical buffer solution containing only 0.2 mM ferrocenemethanol for a repeat EIS scan.

From the Bode and Nyquist plots comparing the same samples at both concentrations, the involvement of ferrocenemethanol in the key processes observed is apparent from the impact on the arcs and impedance at low frequencies that follows the change in concentration (see Figure S5). The observed changes in the EIS plots confirm the attribution of the arc corresponding to the parallel charge transfer resistance (R_{ct}) and double layer CPE (Q_{dl}) equivalence circuit components to the charge transfer process involving FcMeOH, and also that the diffusion limits for the degraded films and uninsulated control samples correspond to the diffusion of FcMeOH as well.

From equivalence circuit fitting, with the 5 times decrease in FcMeOH concentration, R_{ct} increased by roughly 3 times on average, somewhat less than expected, assuming that k_{ET} remains concentration independent (c.f. Equation S3), while σ increased by roughly 4 times on average, also slightly below expectation (c.f. Equation S4).

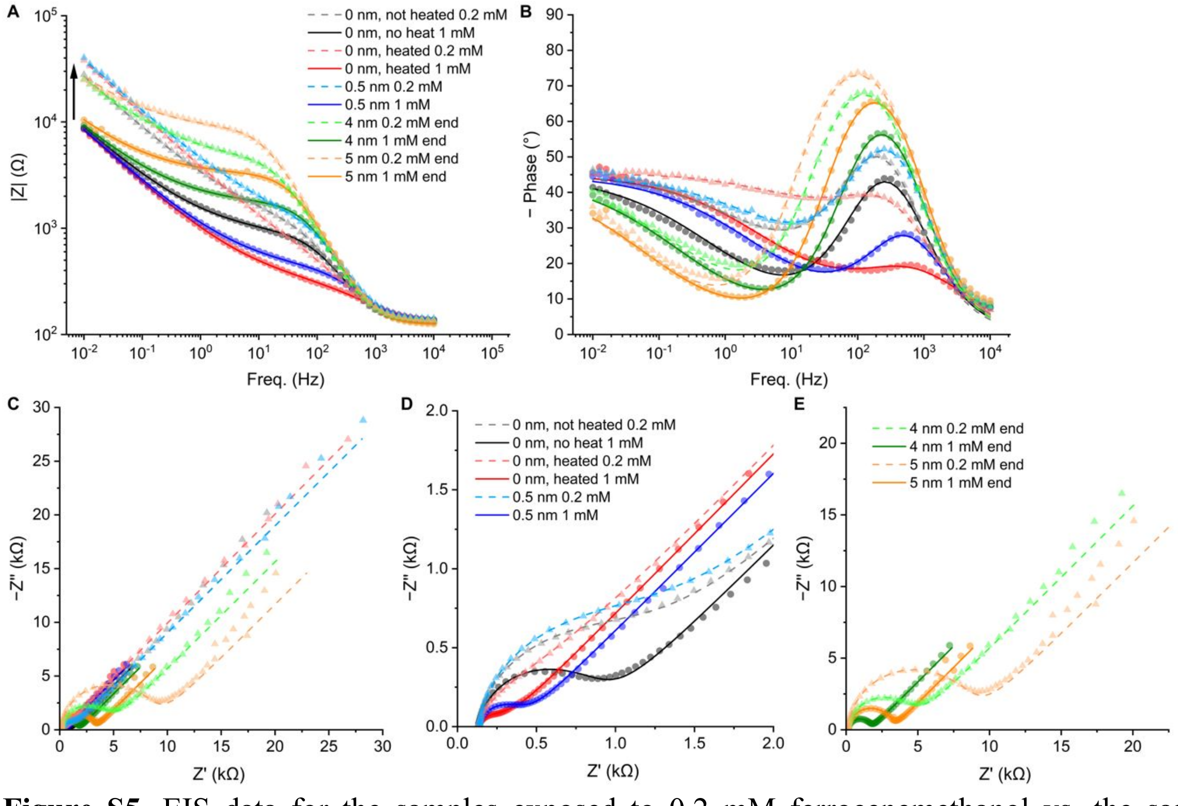

Figure S5. EIS data for the samples exposed to 0.2 mM ferrocenemethanol vs. the same samples with 1.0 mM: (A) Bode magnitude and (B) Bode phase plots; Nyquist plots of (C) all samples, (D) uninsulated samples only (zoomed 15×), and (E) insulated samples only. Solution: 0.1 M pH 7 potassium phosphate buffer.

2.4. EIS Comparisons of 5.0 nm, 4.0 nm and 3.0 nm Insulated Samples over Time

EIS Nyquist Plots – Initial vs. Endpoints for Direct Comparison

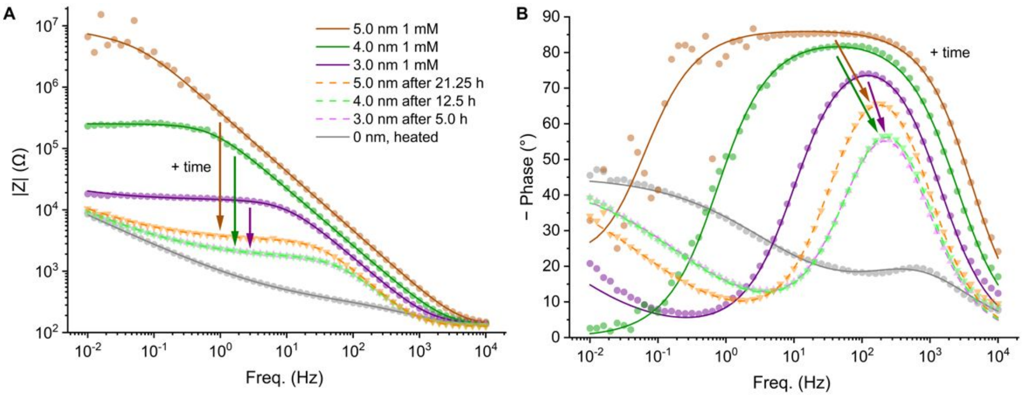

Figure S6. Bode (A) magnitude and (B) Bode phase plots of 0.20 cm^2 ITO glass samples with 5.0 (orange), 4.0 (green) and 3.0 nm (purple) Al_2O_3 layers before (dark, solid lines) and after (light, dashed lines) cycling until stabilisation. Solution: 0.1 M pH 7 potassium phosphate buffer with 1.0 mM FcMeOH. The corresponding Nyquist plots are presented below. Data for uninsulated heated ITO (grey) is shown for comparison.

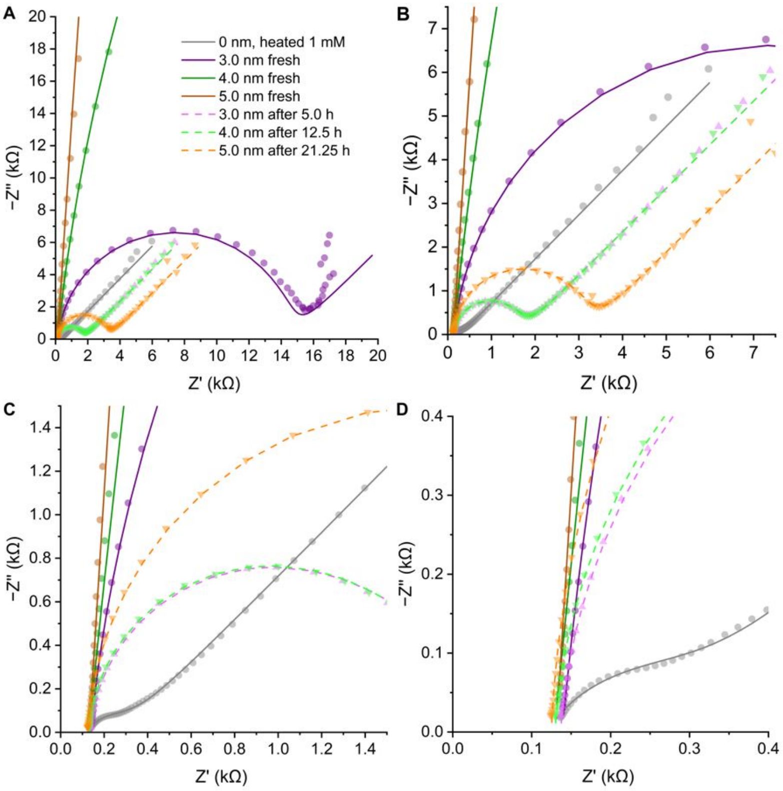

Figure S7. Nyquist plots of 0.20 cm^2 ITO glass samples with 5.0 (orange), 4.0 (green) and 3.0 nm (purple) Al_2O_3 layers before (dark, solid lines) and after (light, dashed lines) cycling until stabilisation. The same data is presented with axes scaled to (A) 20, (B) 7.5, (C) 1.5 and (D) 0.4 kΩ. Solution: 0.1 M pH 7 potassium phosphate buffer with 1.0 mM FcMeOH. Data for uninsulated heated ITO (grey) is shown for comparison.

EIS Nyquist Plots – Timepoints for 5.0 nm, 4.0 nm and 3.0 nm samples

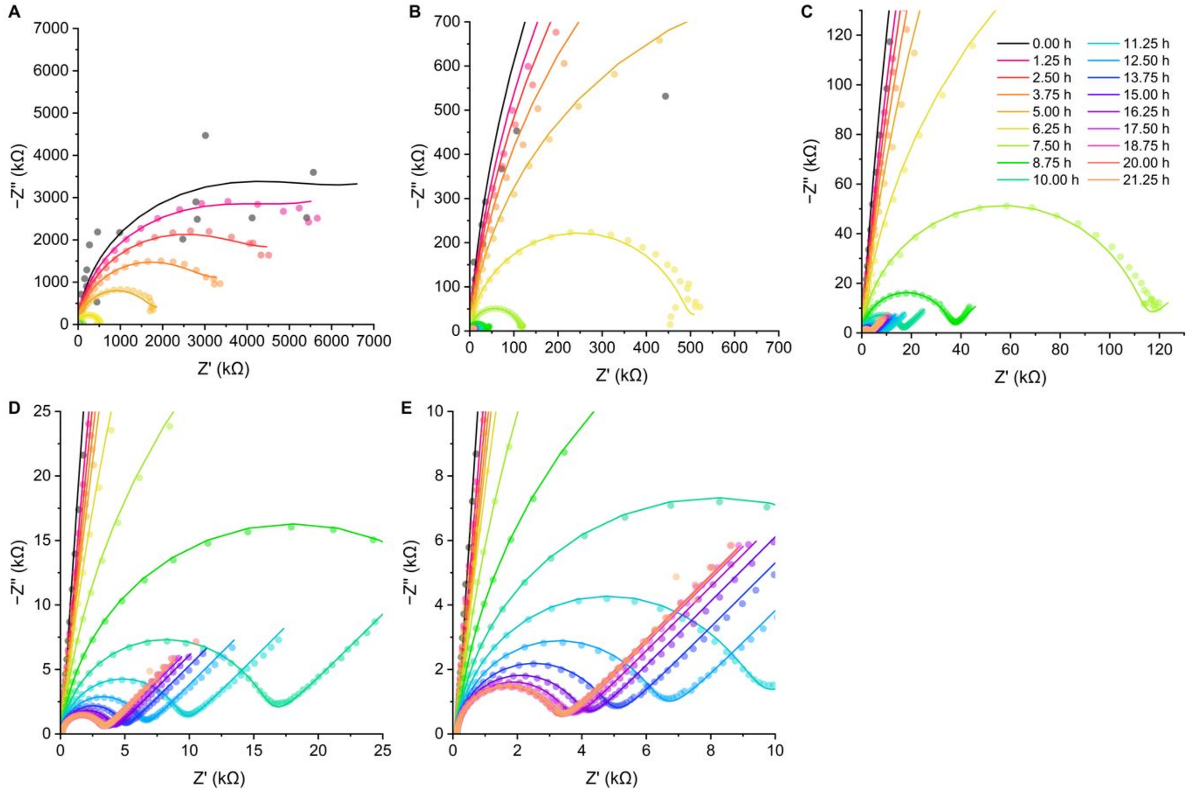

Figure S8. Nyquist plots of 0.20 cm^2 ITO glass with a 5.0 nm Al_2O_3 layer, cycling every 1.25 h until stabilisation. The same data is presented with axes scaled to (A) 7000, (B) 700, (C) 130, (D) 25 and (E) 10 kΩ. Solution: 0.1 M pH 7 potassium phosphate buffer with 1.0 mM FcMeOH.

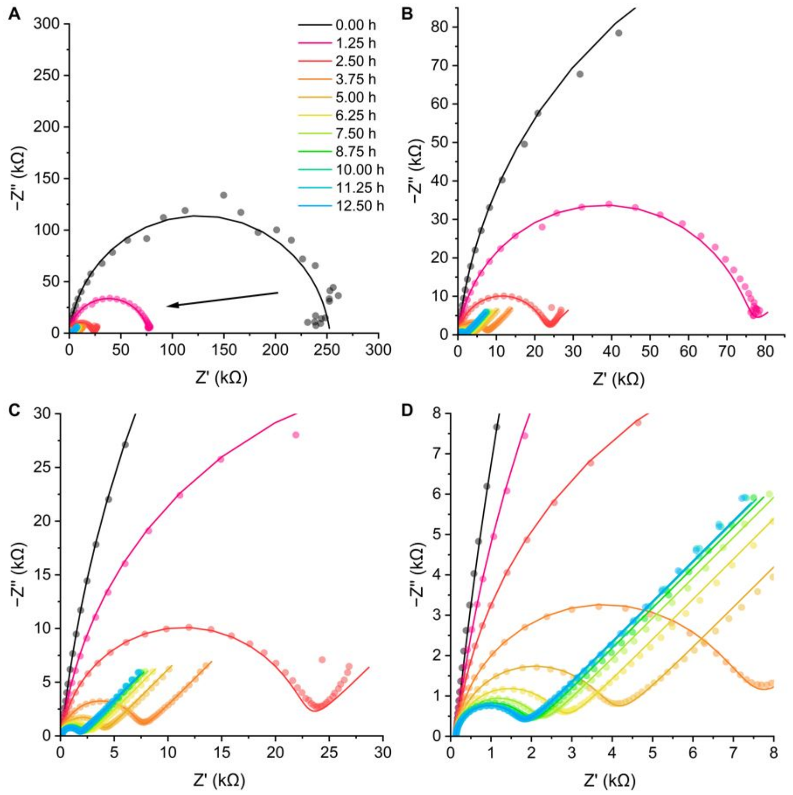

Figure S9. Nyquist plots of 0.20 cm^2 ITO glass with a 4.0 nm Al_2O_3 layer, cycling every 1.25 h until stabilisation. The same data is presented with axes scaled to (A) 300, (B) 85, (C) 30 and (D) 8 kΩ. Solution: 0.1 M pH 7 potassium phosphate buffer with 1.0 mM FcMeOH.

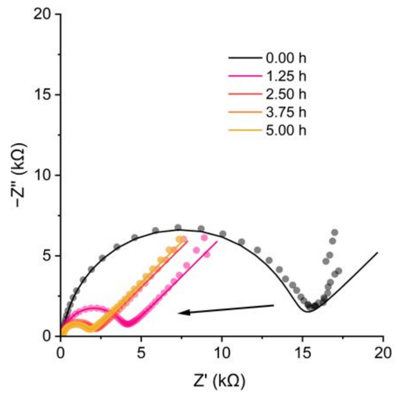

Figure S10. Nyquist plot of 0.20 cm^2 ITO glass with a 3.0 nm Al_2O_3 layer, cycling every 1.25 h until stabilisation. Solution: 0.1 M pH 7 potassium phosphate buffer with 1.0 mM FcMeOH.

Additional Plots of Parameters Extracted from EIS Fitting of 5.0 nm, 4.0 nm & 3.0 nm versus Time

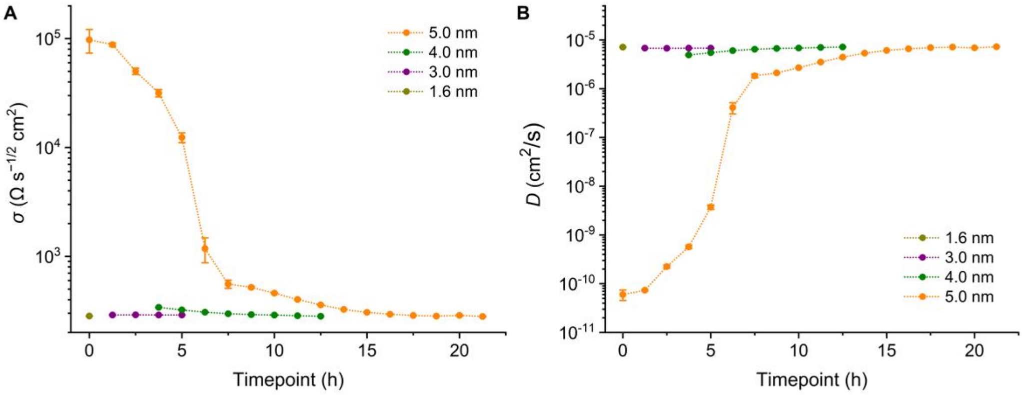

Figure S11. Alternative logarithmic plots of the Warburg coefficients, adjusted for area (left), and derived diffusion coefficients (right), extracted from EIS for 0.20 cm^2 ITO glass samples with 5.0 (orange), 4.0 (green) and 3.0 nm (purple) Al_2O_3 layers, cycling every 1.25 h until stabilisation. 1.6 nm (gold) at 0 h included for comparison. Here, all extracted values for the 5.0 nm sample are shown, although the physicality of values before 7.5 h is questionable. Solution: 0.1 M pH 7 potassium phosphate buffer with 1.0 mM FcMeOH.

Table S7. Fitted Equivalent Circuit Model Parameters for 3 nm, 4 nm and 5 nm Samples at Regular Timepoints over Cycling.

Time	3 nm						
	R_u ($\Omega\cdot cm^2$)	R_{ct} ($\Omega\cdot cm^2$)	Q_{dl} ($\mu S\cdot s^{\alpha}\cdot cm^{-2}$)	α	σ ($\Omega\cdot s^{-1/2}\cdot cm^2$)	k_{ET} ($cm\cdot s^{-1}$)	D ($cm^2\cdot s^{-1}$)
0.00 h	27.2	2820	7.16	0.940	254 *	1.89E-04	8.79E-06 *
1.25 h	27.1	724	9.59	0.946	289	7.36E-04	6.77E-06
2.50 h	26.9	362	10.6	0.950	290	1.47E-03	6.73E-06
3.75 h	26.9	299	11.0	0.953	289	1.78E-03	6.77E-06
5.00 h	26.8	302	11.2	0.957	289	1.77E-03	6.78E-06

Time	4 nm						
	R_u ($\Omega\cdot cm^2$)	R_{ct} ($\Omega\cdot cm^2$)	Q_{dl} ($\mu S\cdot s^{\alpha}\cdot cm^{-2}$)	α	σ ($\Omega\cdot s^{-1/2}\cdot cm^2$)	k_{ET} ($cm\cdot s^{-1}$)	D ($cm^2\cdot s^{-1}$)
0.00 h	25.2	49500	4.74	0.935	18.6 *	1.08E-05	1.65E-03 *
1.25 h	25.4	14800	6.08	0.926	259 *	3.60E-05	8.45E-06 *
2.50 h	25.8	4380	6.82	0.930	311	1.22E-04	5.87E-06
3.75 h	26.0	1380	7.72	0.936	340	3.85E-04	4.91E-06
5.00 h	26.0	727	8.59	0.939	322	7.32E-04	5.47E-06
6.25 h	25.9	490	9.31	0.943	307	1.09E-03	6.03E-06
7.50 h	25.9	385	9.83	0.946	297	1.38E-03	6.43E-06
8.75 h	25.8	335	10.2	0.950	291	1.59E-03	6.68E-06
10.00 h	25.8	309	10.3	0.955	289	1.72E-03	6.81E-06
11.25 h	25.8	302	10.5	0.958	285	1.76E-03	6.99E-06
12.50 h	25.4	303	10.6	0.961	282	1.76E-03	7.15E-06

Time	5 nm						
	R_u ($\Omega\cdot cm^2$)	R_{ct} ($\Omega\cdot cm^2$)	Q_{dl} ($\mu S\cdot s^{\alpha}\cdot cm^{-2}$)	α	σ ($\Omega\cdot s^{-1/2}\cdot cm^2$)	k_{ET} ($cm\cdot s^{-1}$)	D ($cm^2\cdot s^{-1}$)
0.00 h	25.7	1170000	2.27	0.961	97400 *	5.98E-11	4.57E-07 *
1.25 h	24.8	969000	2.70	0.950	87900 *	7.34E-11	5.49E-07 *
2.50 h	24.6	804000	2.89	0.945	50300 *	2.25E-10	6.62E-07 *
3.75 h	24.5	580000	3.12	0.940	31600 *	5.69E-10	9.19E-07 *
5.00 h	24.4	331000	3.36	0.937	12400 *	3.72E-09	1.61E-06 *
6.25 h	24.6	95600	3.63	0.937	1180 *	4.09E-07	5.57E-06 *
7.50 h	25.0	22000	3.96	0.939	556 *	1.83E-06	2.42E-05 *
8.75 h	25.3	6900	4.35	0.944	519	2.11E-06	7.72E-05
10.00 h	25.5	3070	4.89	0.947	459	2.69E-06	1.73E-04
11.25 h	25.5	1780	5.50	0.949	402	3.51E-06	2.99E-04
12.50 h	25.4	1200	6.13	0.952	358	4.43E-06	4.45E-04
13.75 h	25.1	901	6.73	0.955	325	5.36E-06	5.91E-04
15.00 h	24.9	742	7.26	0.958	306	6.07E-06	7.17E-04
16.25 h	24.8	652	7.71	0.961	294	6.57E-06	8.16E-04
17.50 h	24.6	604	8.14	0.964	286	6.93E-06	8.81E-04
18.75 h	24.6	585	8.45	0.967	282	7.11E-06	9.11E-04
20.00 h	24.6	581	8.61	0.971	287	6.90E-06	9.16E-04
21.25 h	24.5	595	9.01	0.971	280	7.23E-06	8.95E-04

* Values extracted before reliable observation of diffusion.

2.5. Original CV Data Used for Stability Assessments in Different Solutions

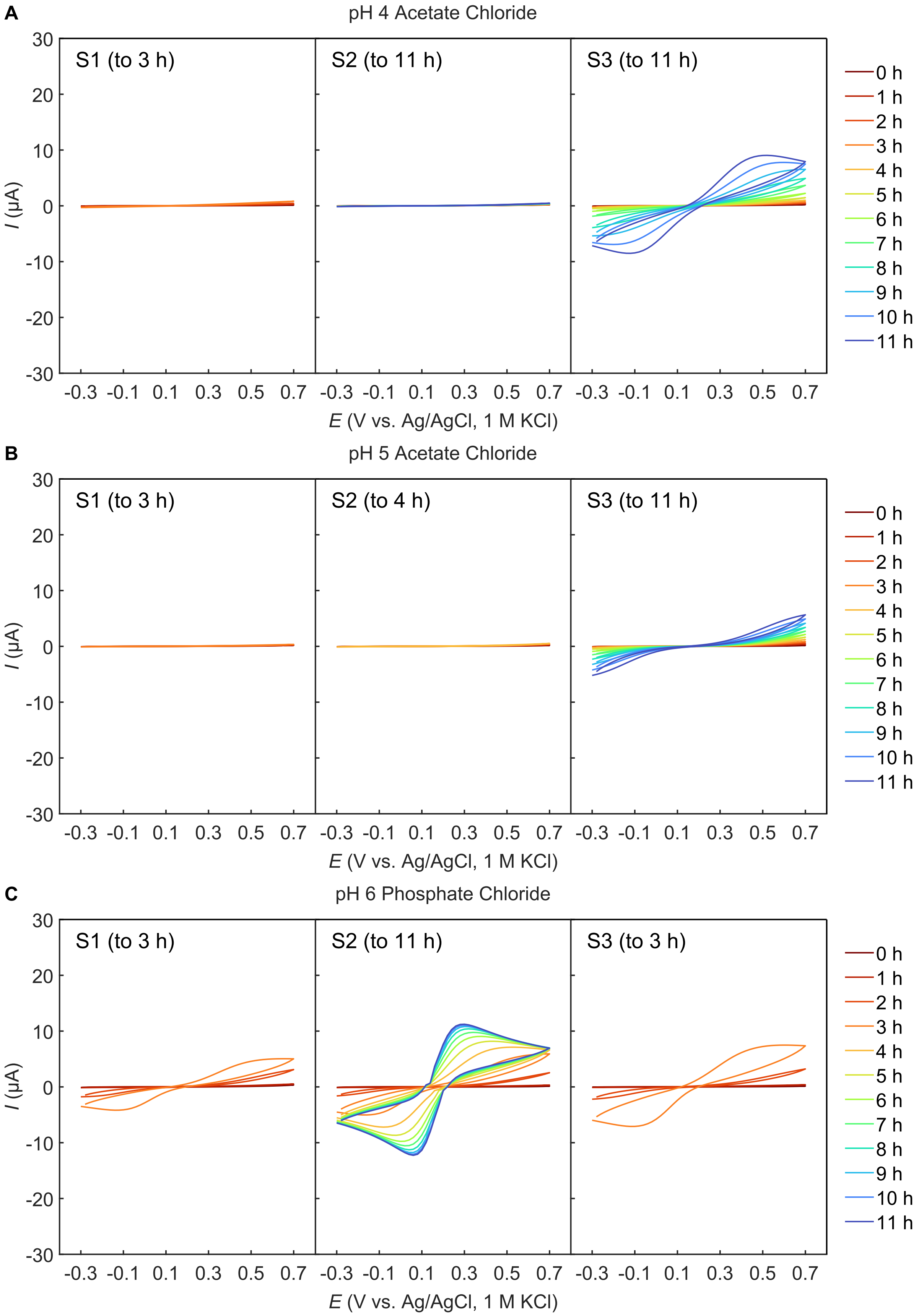

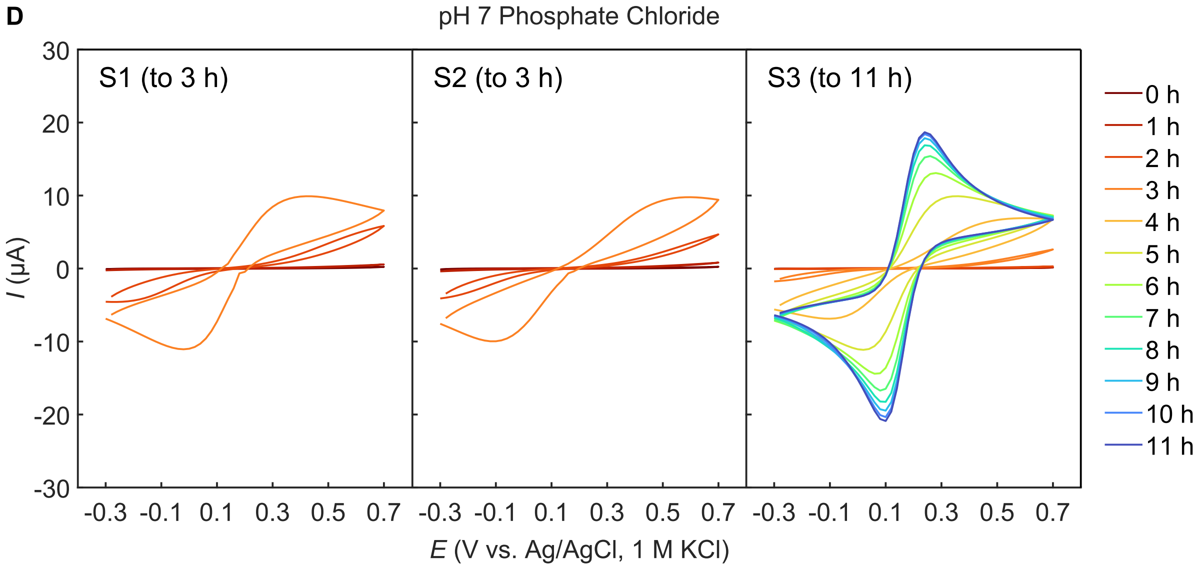
D
pH 7 Phosphate Chloride
S1 (to 3 h)
S2 (to 3 h)
S3 (to 11 h)
I (µA)
E (V vs. Ag/AgCl, 1 M KCl)
0 h
1 h
2 h
3 h
4 h
5 h
6 h
7 h
8 h
9 h
10 h
11 h

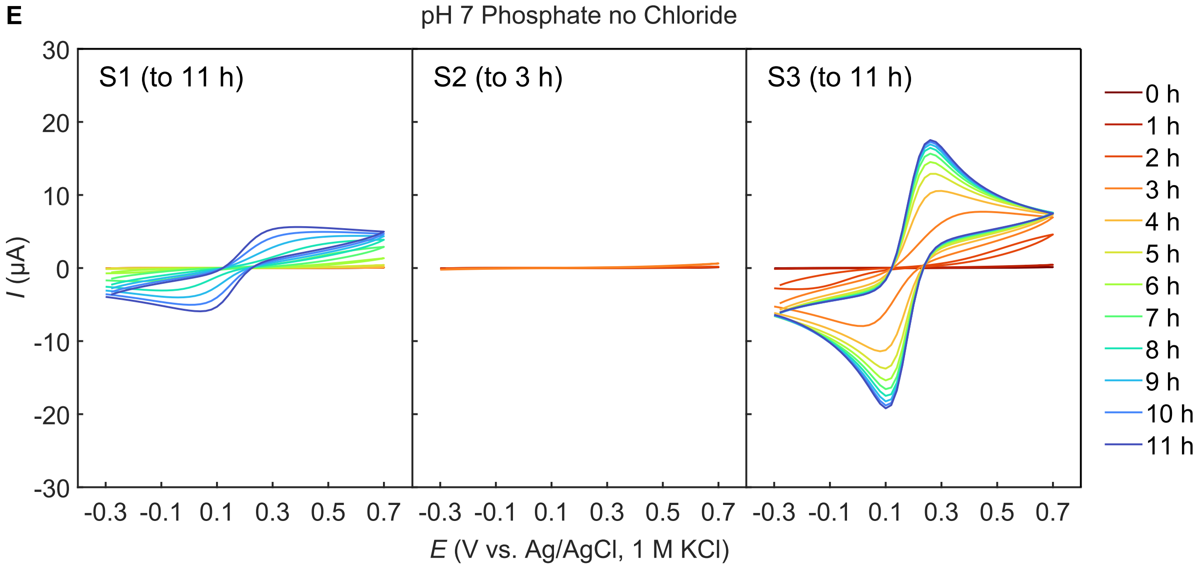
E
pH 7 Phosphate no Chloride
S1 (to 11 h)
S2 (to 3 h)
S3 (to 11 h)
I (µA)
E (V vs. Ag/AgCl, 1 M KCl)
0 h
1 h
2 h
3 h
4 h
5 h
6 h
7 h
8 h
9 h
10 h
11 h

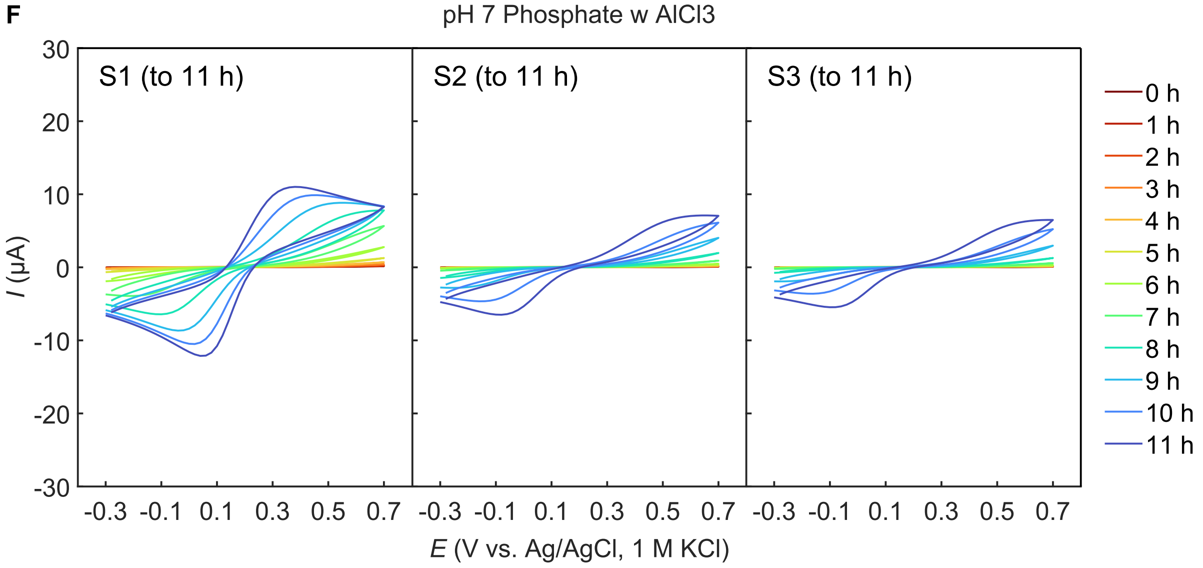
F
pH 7 Phosphate w AlCl3
S1 (to 11 h)
S2 (to 11 h)
S3 (to 11 h)
I (µA)
E (V vs. Ag/AgCl, 1 M KCl)
0 h
1 h
2 h
3 h
4 h
5 h
6 h
7 h
8 h
9 h
10 h
11 h

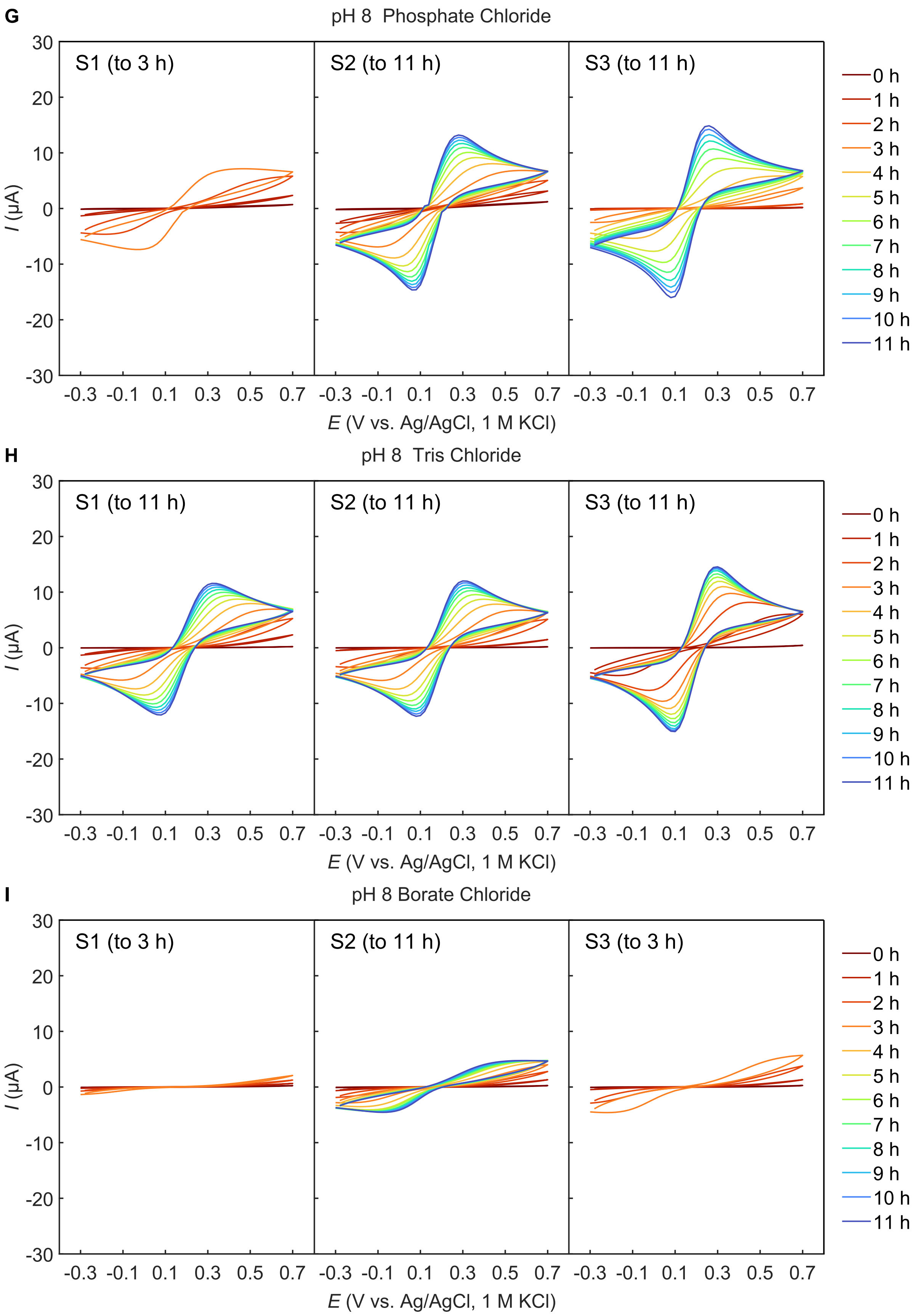
G
pH 8 Phosphate Chloride
S1 (to 3 h)
S2 (to 11 h)
S3 (to 11 h)
H
pH 8 Tris Chloride
S1 (to 11 h)
S2 (to 11 h)
S3 (to 11 h)
I
pH 8 Borate Chloride
S1 (to 3 h)
S2 (to 11 h)
S3 (to 3 h)
I (μA)
30
20
10
0
-10
-20
-30
-0.3 -0.1 0.1 0.3 0.5 0.7
E (V vs. Ag/AgCl, 1 M KCl)
0 h
1 h
2 h
3 h
4 h
5 h
6 h
7 h
8 h
9 h
10 h
11 h

J

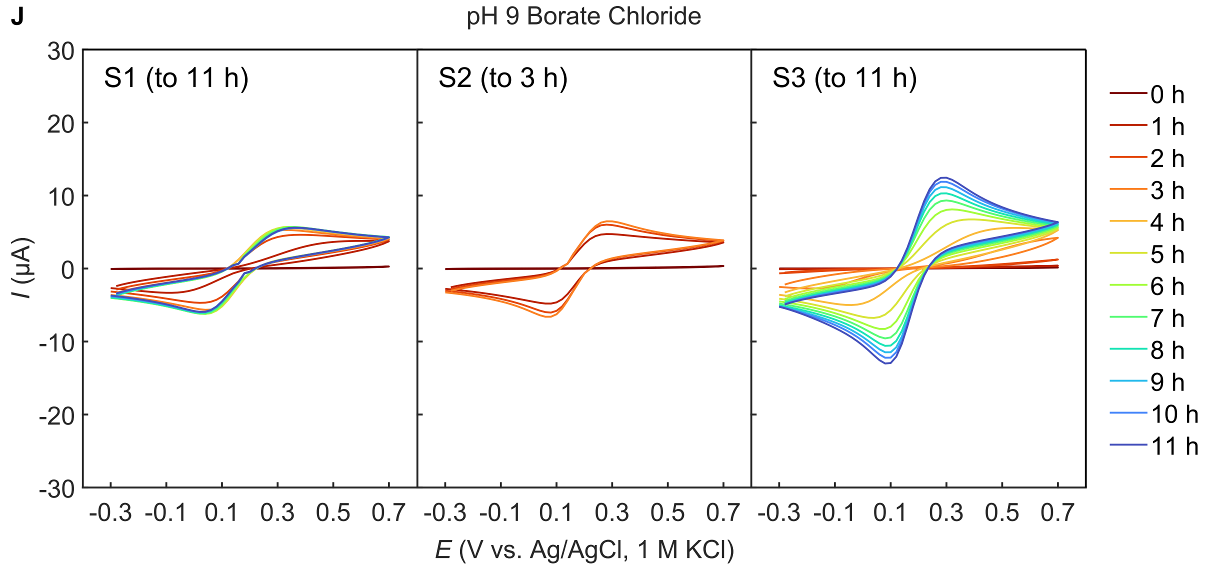

K

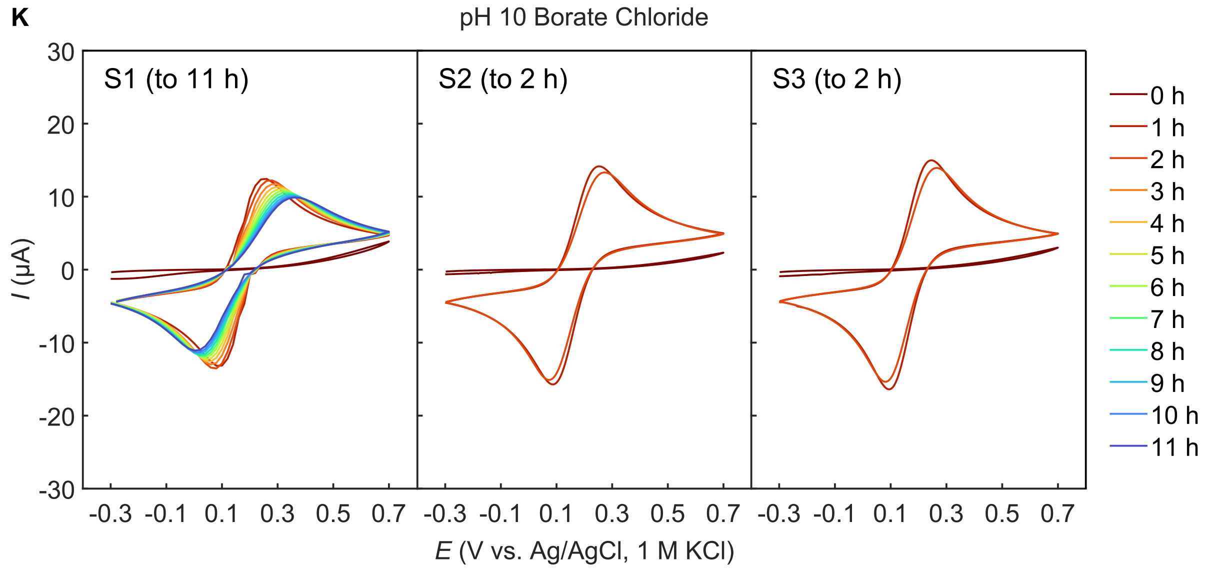

Figure S12. Original CV data taken for triplicate samples of ITO modified with 5.0 nm ALD-Al_2O_3, in different buffers, made up from 0.1 M total concentration of the potassium salt of the conjugate base and the acid, with 0.1 M KCl when chloride was included. Only the voltammograms at each hour timepoint are shown for clarity. Total charge passed and peak currents over time were extracted from these datasets. Solutions tested: (A) pH 4 acetate with chloride; (B) pH 5 acetate with chloride; (C) pH 6 phosphate with chloride; (D) pH 7 phosphate with chloride; (E) pH 7 phosphate, no chloride; (F) pH 7 phosphate with 0.33 mM $AlCl_3$; (G) pH 8 phosphate with chloride; (H) pH 8 tris with chloride; (I) pH 8 borate with chloride; (J) pH 9 borate with chloride; (K) pH 10 borate with chloride.

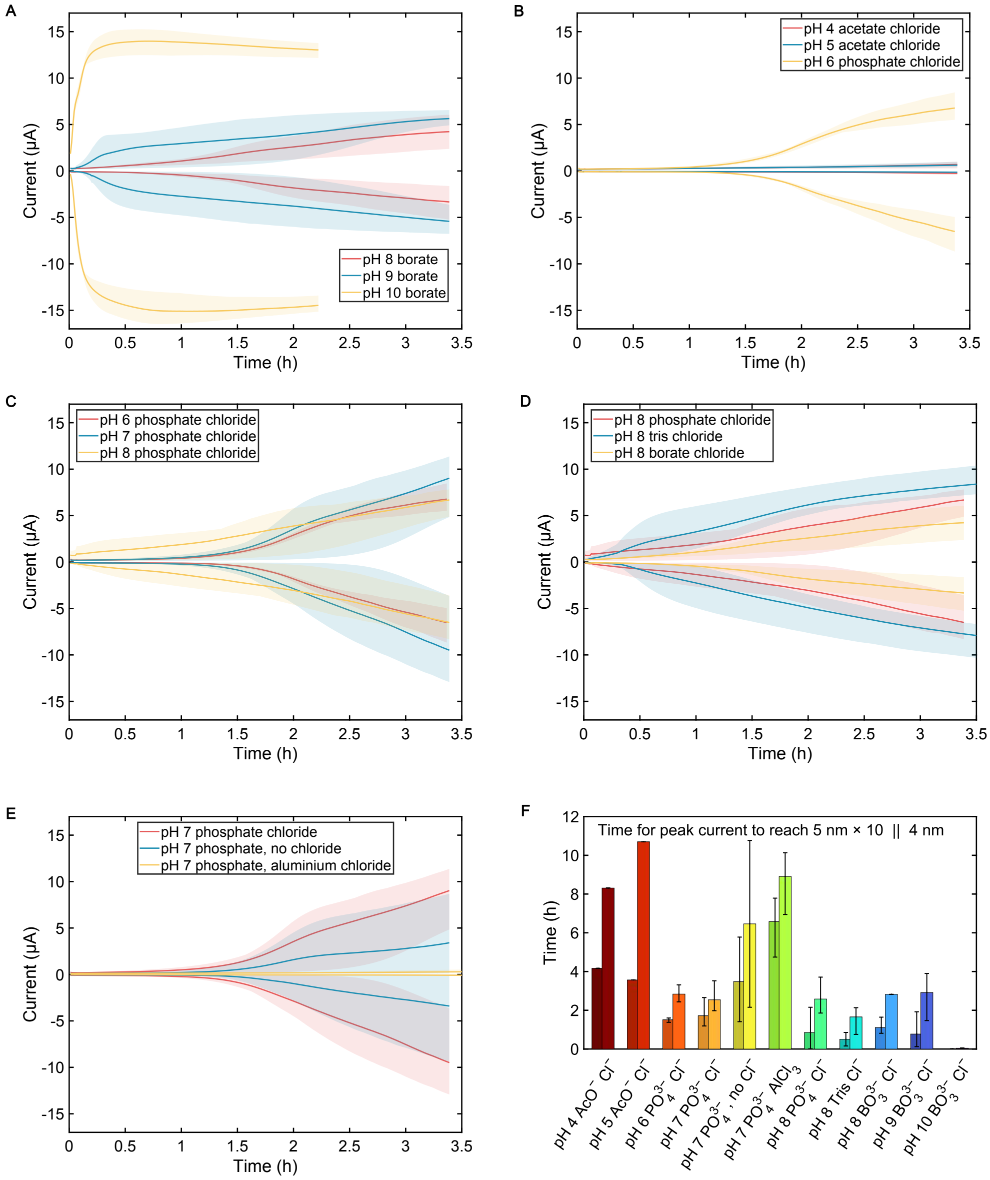

Figure S13. Plots of the peak or otherwise maximum currents of each cycle, against time for the first 3.5 h. The shaded area represents the range from 3 samples under each condition, with the darker line representing the mean as a guide to the eye. Datasets are selected to directly compare: (A) Basic conditions in borate (pH 8, 9, 10); (B) Mild acidic conditions (acetate pH 4 & 5 and phosphate pH 6); (C) Neutral conditions in phosphate (pH 6, 7, 8); (D) Various buffers at pH 8 (phosphate, tris and borate); (E) pH 7 phosphate buffer with 0.1 M KCl, without added chloride, and with 0.33 mM $AlCl_3$. (F) Bar charts showing the average time taken for fresh 5 nm samples to reach the point where their peak anodic current reaches that of fresh 5 nm samples on average (left bars) and that of fresh 4 nm samples on average (right bars). Ranges across triplicates are shown in error bars, except for acetate and pH 8 borate conditions, where only one sample in each case was run long enough to reach these points.

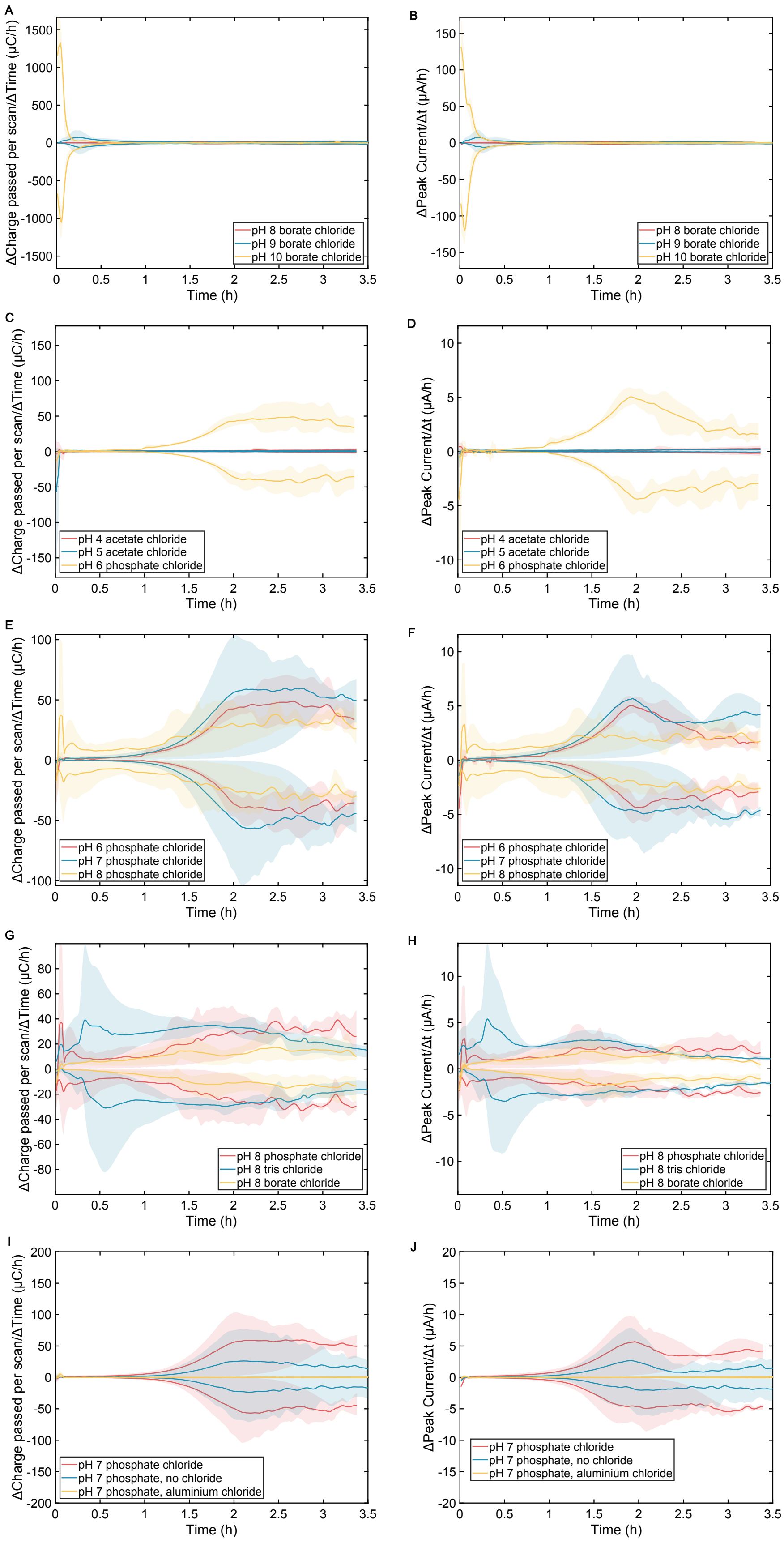

Figure S14. Plots against time of the derivatives over time for the total charge passed per cycle (A,C,E,G,I), corresponding to Fig. 6, and peak currents (B,D,F,H,J) corresponding to Fig. S13.

2.6. Estimate of Moles of Aluminium Deposited

The ratio of Al^{3+} ions in solution to Al atoms in the ALD-Al_2O_3 film was estimated based on previous reports[7] of amorphous phases of Al_2O_3 having densities between **2.1 – 3.6 g/cm³**. Firstly, assuming no precipitation, the amount of Al^{3+} from 0.33 mM $AlCl_3$ added to a 2 mL solution is **660 nmol**. Given the dimensions of an exposed 5 nm film are a cross-sectional area of 2.0×10^{-5} m² and a thickness of 5.0×10^{-9} m, the volume of Al_2O_3 is estimated as **9.8×10^{-14} mm³**. For densities of 2.1 and 3.6 g/cm³, this corresponds to masses of Al_2O_3 of 2.1×10^{-7} and 3.5×10^{-7} g, respectively. From the molar mass of Al_2O_3 of 101.96 g/mol, of which 52.93% is Al, this estimates the amount of Al in the film as between **1.1 – 1.8 nmol**, giving a range of **360 – 620 times more** Al in solution than in the ALD films.

2.7. Further Details on CV Simulations Based on Parameters Extracted from EIS

Simulated CVs for Uninsulated Samples

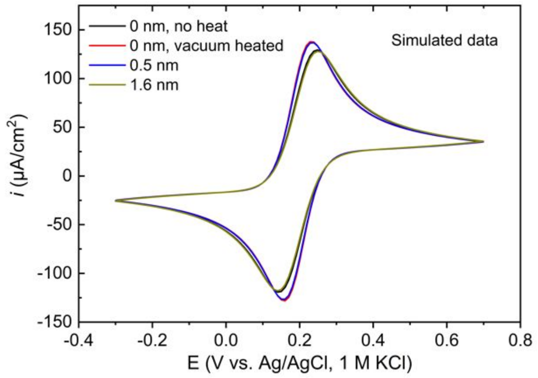

Figure S15. Simulated CV data based on k_{ET} and D values extracted from EIS for the 0.20 cm^2 ITO samples without significant insulating layers, corresponding closely to real data in Main Text Fig. 1.

Simulated CVs with a Fixed Standard Diffusion Coefficient

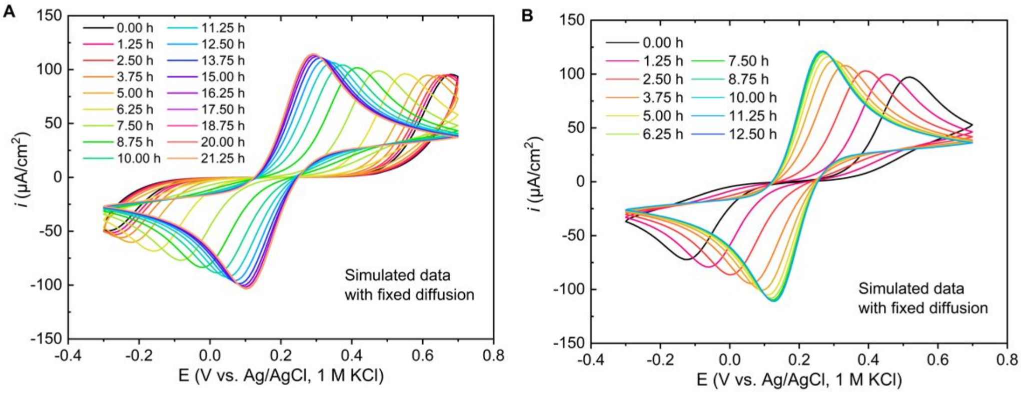

Figure S16. Simulated CV data based on k_{ET} extracted from EIS and a fixed value for D of 6.77 × 10^{-6} $cm^2 \cdot s^{-1}$ for the 0.20 cm^2 ITO samples with (A) 5.0 nm Al_2O_3 and (B) 4.0 nm Al_2O_3 over the EIS measurement timepoints until stabilisation. Solution: 0.1 M pH 7 potassium phosphate buffer with 1.0 mM FcMeOH. D of 6.77 × 10^{-6} $cm^2 \cdot s^{-1}$ is taken from the 0 nm heated ITO sample to be representative of the typical diffusion of 1.0 mM FcMeOH in 0.1 M pH 7 potassium phosphate buffer.

Simulated CVs for 4.0 nm Al_2O_3 over Time

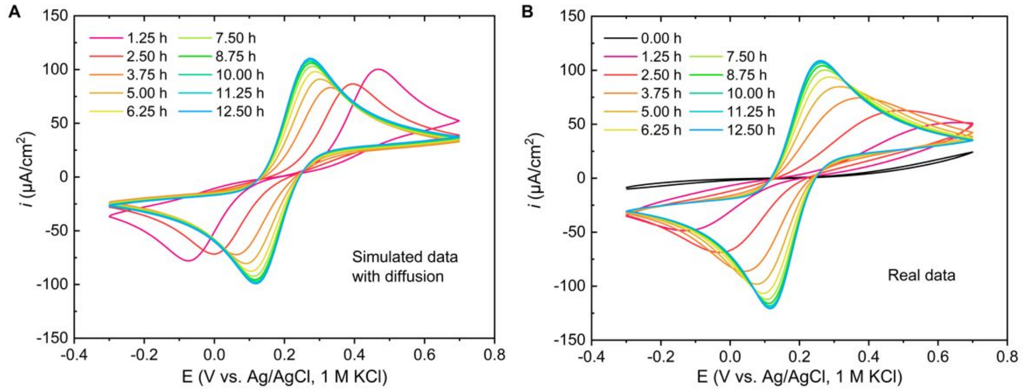

Figure S17. (A) Simulated CV data based on k_{ET} and D values extracted from EIS versus (B) the real CV data recorded before each EIS scan for the 0.20 cm^2 ITO sample with 4.0 nm Al_2O_3 over the EIS measurement timepoints until stabilisation. The 0 h timepoint is excluded due to an unrealistically low extracted D value resulting in an unphysical simulated voltammogram (Table S7). Note that the 1.25 h and 2.50 h timepoints suffer from this issue to a lesser degree. For the other timepoints, taking into account D yields predictions with currents closer to the observed data than when D is fixed (c.f. Fig. S16(B)). Solution: 0.1 M pH 7 potassium phosphate buffer with 1.0 mM FcMeOH.

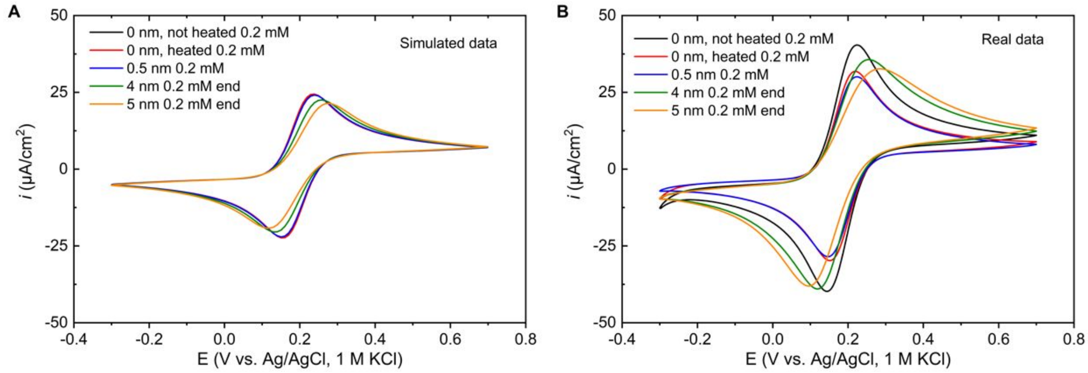

Figure S18. (A) Simulated CV based on EIS data from samples exposed to 0.2 mM FcMeOH *vs.* (B) the real data obtained for the same samples. Solution: 0.1 M pH 7 potassium phosphate buffer.

3. References

(1) Bard, A. J.; Faulkner, L. R. *Electrochemical Methods: Fundamentals and Applications*, 2nd ed.; Wiley: New York, 2001.

(2) Nicholson, R. S. Theory and Application of Cyclic Voltammetry for Measurement of Electrode Reaction Kinetics. *Anal. Chem.* **1965**, *37* (11), 1351–1355. https://doi.org/10.1021/ac60230a016.

(3) Daubert, J. S.; Hill, G. T.; Gotsch, H. N.; Gremaud, A. P.; Ovental, J. S.; Williams, P. S.; Oldham, C. J.; Parsons, G. N. Corrosion Protection of Copper Using Al_2O_3, TiO_2, ZnO, HfO_2, and ZrO_2 Atomic Layer Deposition. *ACS Appl. Mater. Interfaces* **2017**, *9* (4), 4192–4201. https://doi.org/10.1021/acsami.6b13571.

(4) Haeffele, R.; Marcelin, S.; Broussous, L.; Mazet, L.; Normand, B. Electrochemical Characterization of Localized Corrosion Mechanism of ALD Al2O3-Coated Copper for Microelectronic Application. *Corrosion Science* **2024**, *234*, 112135. https://doi.org/10.1016/j.corsci.2024.112135.

(5) Trentin, A.; De L. Gasparini, A.; Faria, F. A.; Harb, S. V.; Dos Santos, F. C.; Pulcinelli, S. H.; Santilli, C. V.; Hammer, P. Barrier Properties of High Performance PMMA-Silica Anticorrosion Coatings. *Progress in Organic Coatings* **2020**, *138*, 105398. https://doi.org/10.1016/j.porgcoat.2019.105398.

(6) Murbach, M.; Gerwe, B.; Dawson-Elli, N.; Tsui, L. Impedance.Py: A Python Package for Electrochemical Impedance Analysis. *JOSS* **2020**, *5* (52), 2349. https://doi.org/10.21105/joss.02349.

(7) Århammar, C.; Pietzsch, A.; Bock, N.; Holmström, E.; Araujo, C. M.; Gråsjö, J.; Zhao, S.; Green, S.; Peery, T.; Hennies, F.; Amerioun, S.; Föhlisch, A.; Schlappa, J.; Schmitt, T.; Strocov, V. N.; Niklasson, G. A.; Wallace, D. C.; Rubensson, J.-E.; Johansson, B.; Ahuja, R. Unveiling the Complex Electronic Structure of Amorphous Metal Oxides. *Proc. Natl. Acad. Sci. U.S.A.* **2011**, *108* (16), 6355–6360. https://doi.org/10.1073/pnas.1019698108.
